# Comparative study between high-fidelity simulation and medium-fidelity simulation in decision-making of nursing students: experimental study*

**DOI:** 10.1590/1518-8345.6847.4269

**Published:** 2024-08-12

**Authors:** Hugo Miguel Santos Duarte, Joana Antunes Castanheira, Ana Sofia Ferreira Pereira, Ângela Pragosa, Edna Tatiana Prazeres Santos, Maria dos Anjos Dixe

**Affiliations:** 1 Instituto Politécnico de Leiria, Escola Superior de Saúde de Leiria, Leiria, LEI, Portugal.; 2 Instituto Politécnico de Leiria, ciTechCare - Center for Innovative Care and Health Technology, Leiria, LEI, Portugal.; 3 Centro Hospitalar Médio Tejo, Hospital de Torres Novas, Torres Novas, TOR, Portugal.; 4 Centro Hospitalar de Leiria, Hospital de Santo André, Leiria, LEI, Portugal.

**Keywords:** Cardiopulmonary Resuscitation, Clinical Decision-Making, Clinical Reasoning, Critical Thinking, High Fidelity Simulation Training, Nursing Students

## Abstract

**Objective:**

to compare the decision-making of Nursing students, before and after theoretical training on basic life support, using the practice of high-fidelity simulation and medium-fidelity simulation.

**Method:**

an experimental study was developed, pre- and post-test type, with quantitative, descriptive and inferential analysis, with theoretical training on basic life support and clinical simulation practices, and with evaluation of knowledge and decision-making of Nursing students, at three different moments - before the simulation scenario (T0), after the simulation scenario (T1) and after clinical teaching (T2).

**Results:**

51 students participated in the research, with an average age of 20.25±3.804, of which 92.2% were female. Statistically significant differences (*F*=6.47; p=0.039) were evident regarding the definition of the problem and development of objectives in decision-making in the experimental group.

**Conclusion:**

Nursing students demonstrate an adequate level of knowledge and a good decision-making process, based on the most current instruments produced by scientific evidence, in clinical simulation scenarios in basic life support, and this innovative methodology should be deepened in the Nursing teaching.

## Highlights

**(1)** Clinical simulation promotes good decision-making of Nursing students.

**(2)** Students demonstrated adequate knowledge about basic life support.

**(3)** Knowledge and practice define the fidelity of clinical simulation.

**(4)** Basic life support can be developed by high-fidelity simulation.

## Introduction

Nursing teaching is fundamentally supported by two main methodologies: Traditional Teaching Method (TTM) and Innovative Teaching Method (ITM)^1^ ^)-(^ ^2^. The first is based on the simple transmission of knowledge between the teacher and the students, while ITM is characterized by the interaction between the student’s previous knowledge and the new knowledge acquired, leading to greater cognitive stability^1^ ^)-(^ ^3^.

Within teaching methodologies, there are several learning tools. The learning process in Nursing must be supported by pedagogical strategies framed in the statute of student centrality, allowing the mobilization of their previous knowledge, not just assimilating the knowledge transmitted, as well as enhancing the development of autonomy, critical thinking and clinical judgment in the Decision-Making (DM) process, highlighting High-Fidelity Simulation (HFS) for this purpose^1^ ^)-(^ ^2^ ^),(^ ^4^ ^)-(^ ^5^.

HFS, one of the types of Clinical Simulation (CS) included in the ITM, is a learning strategy that integrates theoretical knowledge with clinical practice, and which privileges reflection and critical thinking among Nursing students, and is also used to stimulate analysis of problems from multiple perspectives, contributing to the improvement of clinical reasoning and the DM process within the scope of technical and non-technical skills^3^ ^)-(^ ^4^ ^),(^ ^6^ ^)-(^ ^10^.

Understanding the concept of DM is an important factor in Nursing education, so that skills can be developed in the areas of problem solving, communication, prioritization, clinical reasoning, clinical judgment and critical thinking^11^ ^)-(^ ^12^.

Clinical DM is based on three fundamental requirements: knowledge in the area of activity; thinking skills; and an adequate perception of the situation or problem^11^.

Furthermore, DM is a complex mental process, which must include the following steps: identification and definition of the problem; development of objectives^13^; search for data/facts^12^ ^)-(^ ^13^; development of a model^13^; evaluation of alternatives and selection of the best solution^12^ ^)-(^ ^13^; and implementation of the decision or course of action planning^3^ ^),(^ ^13^. Authors^14^ also state that the DM process includes four main phases: data collection; data processing; action planning; and implementation of the plan^14^.

Given the importance of adequate DM in clinical practice and the need to understand whether there are differences in Nursing teaching carried out using two tools, included in the ITM, this study aims to answer the research question: Are there differences between the decision-making of Nursing students, before and after theoretical training on Basic Life Support (BLS), using the practice of high-fidelity simulation and medium-fidelity simulation? In this sense, the following main objective was defined: to compare the decision-making of Nursing students, before and after theoretical training on basic life support, using the practice of high-fidelity simulation and medium-fidelity simulation.

## Method

### Study design

Experimental study, pre- and post-test type, designed with control group and blinding, with data collection carried out between February 2020 and February 2021.

To carry out this study, variables were considered to allow for a sociodemographic and academic characterization of the participants (gender, age and academic year), and to evaluate the knowledge and DM of Nursing students on BLS. Nursing students’ knowledge about BLS was assessed using an instrument composed of 37 closed-answer true and false questions, based on a public instrument that allows scores between 0 and 37 points and on the most recent guidelines on BLS^15^ ^)-(^ ^16^. The evaluation of participants’ DM on BLS was carried out using the Clinical Decision-Making in Nursing Scale^©^ (CDMNS-PT^©^)^17^, translated and cross-culturally validated for Portuguese Nursing students, consisting of 23 items, each of them answered on an ordinal frequency scale that varies from 1 to 5 (1-Never and 5-Always), with scores ranging between 23 and 115 points, with higher scores reflecting higher DM perception rates. The CDMNS-PT^©^ is composed of three factors: Factor 1 - Definition of the Problem and Development of the Objectives (F1); Factor 2 -Search and Data Processing (F2); and Factor 3 - Evaluation of Alternatives, Planning and Implementation of Action (F3)^17^.

### Study location

The study was implemented at the *Escola Superior de Enfermagem de Coimbra* - Portugal.

### Participants

**Figure 1:**
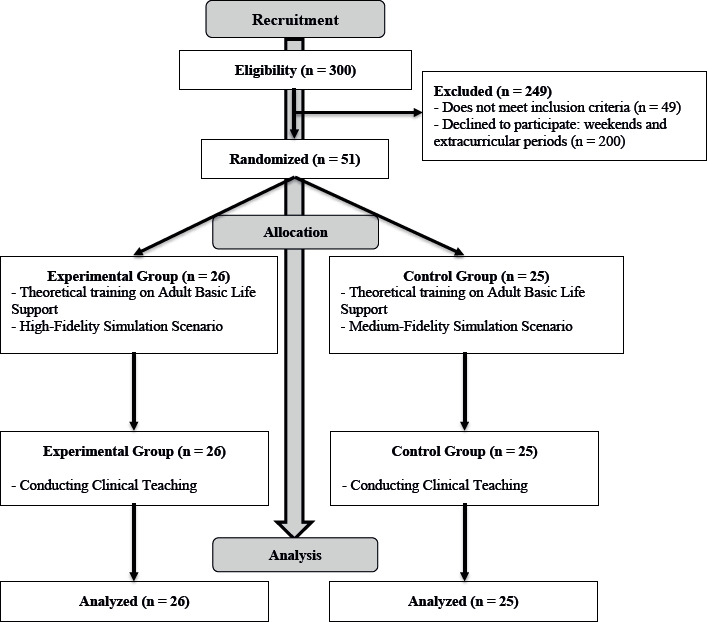
CONSORT* Flowchart *CONSORT = Consolidated Standards of Reporting Trials

The accessible population (N) was 300 Nursing students from that school. The inclusion criteria for participants were not to have previous experience in HFS practices, not to have carried out clinical practices, not to have provided healthcare before starting the degree course, not to have certified training in BLS, and to have no experience working in a real situation of BLS. All students who presented a positive response to these situations were excluded from the study. Thus, of the accessible population (N=300), 100 students volunteered (33%), but of these only 51 met the inclusion criteria (Figure 1). The loss of volunteers is related to the fact that the theoretical training took place on the weekend, during an extracurricular period.

The sample calculation was carried out, based on the population size (N=300), with a confidence level of 90% and a margin of error of 11%, for a sample equal to or greater than 48 students. It should be noted that there was no sample loss between the different moments of variable evaluation.

### Interventions

**Figure 2:**
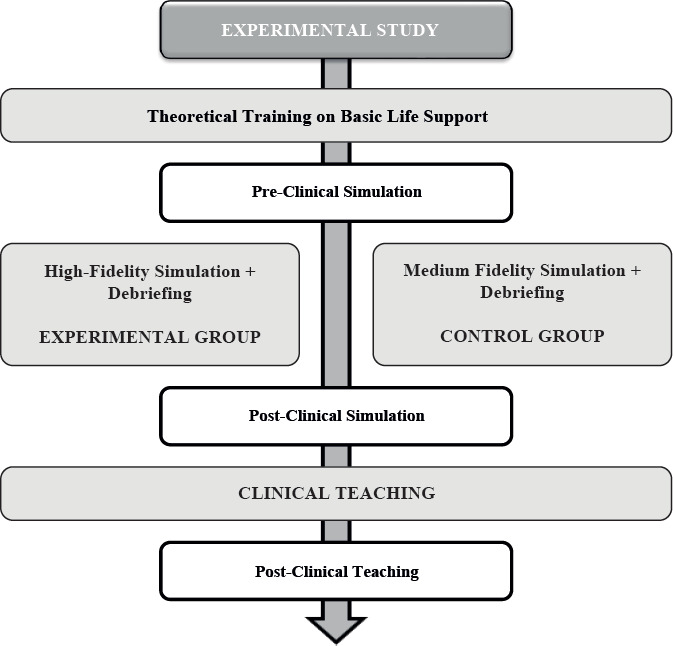
Experimental study and its interventions

After completing Informed Consent, students were randomized into Experimental Group (EG) and Control Group (CG). They were also provided with a training guide to study health care applied in BLS practice. The following week, participants received theoretical training on BLS, in an extracurricular period, lasting approximately 45 minutes, taught by a Medical-Surgical Nursing Specialist Nurse, with certified training in BLS by the American Heart Association (Figure 2).

After completing the theoretical training, the students completed a questionnaire, which allowed to carry out sociodemographic and academic characterization, assess their level of knowledge about BLS^15^ ^)-(^ ^16^ and evaluate their DM in BLS^17^, this being the evaluation moment before carrying out the scenario (T0). The questionnaires were administered by researchers, who did not participate as instructors of the CS scenarios.

The 51 Nursing students were then divided, respecting the randomization carried out initially, into two groups, using *Resusci Anne Laerdal* ^*®*^ in the EG - HFS - Group 1, and *MegaCode Kelly Laerdal* ^*®*^ simulators in the CG - Medium-Fidelity Simulation (MFS) - Group 2, and carrying out the scenarios on BLS.

The EG, before carrying out the HFS scenario, had a prebriefing with an explanation of: what CS is; what the HFS laboratory contains; what will be presented to them inside the HFS laboratory; and what is the objective of practicing the HFS scenario. The same situation was stated with the CG, but in this case about MFS. Then, within each group, participants entered their respective laboratory in pairs, where they had 6 minutes to perform the BLS procedure, in an in-hospital environment, starting with a conscious patient with chest pain, culminating in cardiorespiratory arrest. Throughout the HFS scenarios there was one instructor inside the laboratory and another inside the control room, while in the MFS scenarios there was only one instructor inside the laboratory. Immediately after the scenarios, a session of reflective debriefing^18^ took place between each pair of participants and instructor, and a new questionnaire was completed, in order to reassess the students’ knowledge and DM about BLS after the conclusion of the scenarios (T1). Clinical Teaching (CT) was then developed for approximately 4 months, after which the students completed a third questionnaire, with a new evaluation of the level of knowledge and DM on BLS, this being considered the evaluation moment after carrying out CT (T2).

### Outcomes

The primary outcomes of this study predicted the existence of statistically significant differences between the level of knowledge about BLS of Nursing students and the research groups, as well as the existence of statistically significant differences between DM in BLS practices of Nursing students and the research groups.

### Randomization

The students were randomized, using the white ball/black ball system, into EG and CG, forming pairs within each of these groups assigned in the order of randomization, immediately after completing the Free and Informed Consent to participate in this study.

### Blinding

Randomization was carried out by the main investigator, and was only known to him and the instructors involved in the practical BLS training. None of the Nursing students were aware of the correspondence between the white ball/black ball and the respective group.

### Data analysis

Data processing was carried out using the *Statistical Package for the Social Sciences* program, version 26.0. With regard to descriptive statistics techniques, absolute and relative frequencies, measures of central tendency and measures of dispersion and variability were calculated. Non-parametric paired and interdependent tests were also applied - Mann-Whitney and Friedman. For statistical tests, significance levels were used: p > 0.05 - non-significant difference; and p ≤ 0.05 - significant difference^19^. The interpretation of the effect size results took into account the values: r ≤ 0.2 - small effect size; 0.2 < r ≤ 0.5 - medium effect size; 0.5 < r ≤ 1 - high effect size; and r > 1 - very high effect size^19^.

### Ethical aspects

The research protocol was approved by a Health Sciences Ethics Committee, with Opinion Number P.625-11/2019. All data collected, after obtaining Free and Informed Consent from the participants, was treated with the confidentiality and anonymity required by the rigor and standards of the investigation.

## Results

51 Nursing students participated in this study, with an average age of 20 years (20.3±3.8), of which 92.2% were female. Before carrying out the CS scenarios (T0), using Levene’s test statistics, no statistically significant differences were found between the EG and CG in the variables age (313; p=0.786), knowledge (226; p=0.055 ) and DM (265; p=0.258), demonstrating the homogeneity of the sample in relation to these data.

Regarding the BLS knowledge variable, and despite the students presenting a good level of knowledge, we found that over time there was a decrease in the average score in both study groups (Table 1).

**Table 1:** Characterization of knowledge and decision-making of Nursing students and differences between research groups in the three evaluation moments. Coimbra, Portugal, 2021

Variable			Group	M*	SD^†^	MR^‡^	U^§^	*p* ^*||*^
**Knowledge**		T0^¶^	EG**	33.2	1.6	29.8	226	0.055
			CG^††^	32.3	1.5	22.0		
		T1^‡‡^	EG**	32.6	1.7	25.0	300	0.631
			CG^††^	32.7	1.6	27.0		
		T2^§§^	EG**	30.9	1.8	24.4	284.5	0.440
			CG^††^	31.3	2.1	27.6		
								
**Decision-making**	T^||||^	T0^¶^	EG**	95.6	7.2	23.7	265	0.258
			CG^††^	98.3	7.9	28.4		
	F1^¶¶^		EG**	49.4	4.0	23.1	250.5	0.159
			CG^††^	51.2	4.6	29.0		
	F2***		EG**	20.7	2.5	24.5	285	0.447
			CG^††^	21.2	2.5	27.6		
	F3^†††^		EG**	25.5	2.5	24.8	294	0.555
			CG^††^	25.8	2.6	27.2		
							
	T^||||^	T1^‡‡^	EG**	95.3	8.4	23.4	258	0.206
			CG^††^	98.2	6.9	28.7		
	F1^¶¶^		EG**	49.5	4.6	23.5	260.5	0.222
			CG^††^	50.9	3.7	28.6		
	F2***		EG**	20.9	2.6	24.7	292	0.530
			CG^††^	21.4	2.5	27.3		
	F3^†††^		EG**	24.9	3.3	24.5	287	0.470
			CG^††^	25.9	2.6	27.5		
							
	T^||||^	T2^§§^	EG**	95.3	8.7	25.5	312.5	0.814
			CG^††^	95.5	7.9	26.5		
	F1^¶¶^		EG**	51.0	4.6	26.2	319	0.910
			CG^††^	50.8	4.6	25.8		
	F2***		EG**	19.8	2.8	26.9	301.5	0.655
			CG^††^	19.4	2.6	25.1		
	F3^†††^		EG**	24.5	3.9	25.0	299	0.621
			CG^††^	25.3	2.9	27.0		

*M = Mean; ^†^DP = Standard Deviation; ^‡^MR = Mean Rank; ^§^U = Mann-Whitney Test; ^||^p = Significance; ^¶^T0 = Evaluation moment before carrying out the scenario; **EG = Experimental Group; ^††^CG = Control Group; ^‡‡^T1 = Evaluation moment after carrying out the scenario; ^§§^T2 = Evaluation moment after clinical teaching; ^||||^T = Total Scale Sum; ^¶¶^F1 = Factor 1 - Definition of the Problem and Development of the Objectives; ^***^F2 = Factor 2 - Search and Data Processing; ^†††^F3 = Factor 3 - Evaluation of Alternatives, Planning and Implementation of Action

Regarding the DM of Nursing students within the scope of the BLS, a variable analyzed by applying the CDMNS-PT^©^, there is an oscillation of medians between 94 and 100 points in the entire instrument, in both research groups, in the three evaluation moments. Within the EG, there is a maintenance of the DM level of Nursing students, in this case using the HFS, while in the CG, using the MFS, there is a decrease in the score throughout the three evaluation moments, more evident in the third moment, after carrying out the CT.

Taking into account the moments of evaluation and the differences between groups, when we consider the variable level of knowledge and DM of Nursing students, it is observed that there tend to be no statistically significant differences between the various evaluation moments of each of the variables and the research groups - EG and CG.

In longitudinal terms, it is observed that DM presents statistically significant differences in factor 1 *Definition of the Problem and Development of the Objectives*, compared to the EG (p < 0.05), and in factor 2 *Search and Data Processing* in the CG (p < 0.01). The overall value of DM presents non-significant differences in the two research groups, within the scope of the various evaluation moments. In terms of the level of knowledge, statistically significant differences (p < 0.01) emerge in both research groups, between the three evaluation moments, being more significant in the EG (p < 0.001) (Table 2).

**Table 2:** Results of applying the Friedman test on the level of knowledge and decision-making of Nursing students according to the research group in the three evaluation moments. Coimbra, Portugal, 2021

Group	Variable			MR*	M^†^	SD^‡^	*F* ^*§*^	*p* ^*||*^
**Control group** (n^¶^=25)	**Knowledge**		T0**	2.0	32.3	1.5	11.04	0.004
			T1^††^	2.4	31.3	2.0		
			T2^‡‡^	1.6	31.3	2.1		
							
	**Decision-making**	T^§§^	T0**	2.2	98.3	7.9	5.71	0.058
			T1^††^	2.2	98.2	6.9		
			T2^‡‡^	1.6	95.5	7.9		
						
		F1^||||^	T0**	2.0	51.2	4.6	0.02	0.989
			T1^††^	2.0	50.9	3.7		
			T2^‡‡^	2.0	50.8	4.5		
						
		F2^¶¶^	T0**	2.2	21.2	2.5	11.04	0.004
			T1^††^	2.3	21.4	2.5		
			T2^‡‡^	1.5	19.4	2.6		
						
		F3***	T0**	2.1	25.8	2.6	0.58	0.750
			T1^††^	2.0	25.9	2.6		
			T2^‡‡^	1.9	25.3	2.9		
								
**Experimental group** (n^¶^=26)	**Knowledge**		T0**	2.5	33.2	1.6	19.49	0.001
			T1^††^	2.1	31.5	1.7		
			T2^‡‡^	1.4	30.9	1.8		
							
	**Decision-making**	T^§§^	T0**	2.1	95.6	7.2	0.55	0.758
			T1^††^	2.0	95.3	8.4		
			T2^‡‡^	1.9	95.3	8.7		
						
		F1^||||^	T0**	1.8	49.4	4.0	6.47	0.039
			T1^††^	1.8	49.6	4.6		
			T2^‡‡^	2.4	51.0	4.6		
						
		F2^¶¶^	T0**	2.2	20.7	2.5	5.61	0.061
			T1^††^	2.2	20.9	2.6		
			T2^‡‡^	1.7	19.8	2.8		
						
		F3***	T0**	2.2	25.5	2.5	1.33	0.515
			T1^††^	1.9	24.9	3.3		
			T2^‡‡^	1.9	24.5	3.9		

*MR = Mean Rank; ^†^M = Mean; ^‡^SD = Standard Deviation; ^§^F = Friedman Test; ^||^p = Significance; ^¶^n = Sample; **T0 = Evaluation moment before carrying out the scenario; ^††^T1 = Evaluation moment after carrying out the scenario; ^‡‡^T2 = Evaluation moment after clinical teaching; ^§§^T = Total scale sum; ^||||^F1 = Factor 1 - Definition of the Problem and Development of the Objectives; ^¶¶^F2 = Factor 2 - Search and Data Processing; ***F3 = Factor 3 - Evaluation of Alternatives, Planning and Implementation of Action

Due to the existence of statistically significant differences in the Definition of the Problem and Development of the Objectives (F1) of the DM variable, compared to the EG, and in Search and Data Processing (F2), of the same variable, compared to the CG, a more in-depth analysis of these factors was carried out. In this way, it is observed that there are statistically significant differences between the F1 of the DM of EG Nursing students and the value presented between T0 and T2 (p = 0.010). With regard to the F2 of the DM, compared to the CG, it also reveals statistically significant differences at moments T0 and T2 (p = 0.003) and at moments T1 and T2 (p = 0.001) (Table 3).

**Table 3:** Results of the application of the Wilcoxon signed-rank test on the evaluation moments of knowledge and decision-making of Nursing students according to the research group. Coimbra, Portugal, 2021

Group	Variable			NC*	PC^†^	D^‡^	Z^§^	*p* ^*||*^	*Effect Size*
**Experimental group** (n^¶^=26)	**Knowledge**		T0**	14	4	8	-2.29	0.022	0.317
			T1^††^						
			T1^††^	18	5	3	-3.02	0.003	0.418
			T2^‡‡^						
			T0**	21	3	2	-3.52	0.000	0.488
			T2^‡‡^						
									
**Control group** (n^¶^=25)			T0**	2	11	12	-2.07	0.039	0.292
			T1^††^						
			T1^††^	16	4	5	-2.85	0.004	0.402
			T2^‡‡^						
			T0**	13	6	6	-2.27	0.023	0.322
			T2^‡‡^						
									
**Experimental group** (n^¶^=26)	**Decision-making**	F1^§§^	T0**	10	12	4	-0.41	0.680	---
			T1^††^						
			T1^††^	7	18	1	-1.60	0.111	---
			T2^‡‡^						
			T0**	6	15	5	-2.58	0.010	0.506
			T2^‡‡^						
									
**Control group** (n^¶^=25)		F2^||||^	T0**	7	8	10	-0.84	0.399	---
			T1^††^						
			T1^††^	16	3	6	-3.24	0.001	0.457
			T2^‡‡^						
			T0**	14	4	7	-3.02	0.003	0.427
			T2^‡‡^						

*NC = Negative Classifications; ^†^PC = Positive Classifications; ^‡^D = Draws; ^§^ Z = Wilcoxon Signed Rank Test; ^||^p = Significance; ^¶^n = Sample; **T0 = Evaluation moment before carrying out the scenario; ^††^T1 = Evaluation moment after carrying out the scenario; ^‡‡^T2 = Evaluation moment after clinical teaching; ^§§^F1 = Factor 1 - Definition of the Problem and Development of the Objectives; ^||||^F2 = Factor 2 - Search and Data Processing

In view of these results, where three moments with statistically significant differences were identified, within them the improvements that occurred in terms of DM of Nursing students were analyzed, denoting the evolution from T0 to T2 of the EG, with evidence of 15 classifications with improved DM. The effect size was calculated, identifying values between 0.427 and 0.457 in Search and Data Processing (F2), and 0.506 in Definition of the Problem and Development of the Objectives (F1), which highlights a high effect size in the last one^19^.

As differences in the level of knowledge were identified over time, it was verified whether there were differences between the total knowledge of Nursing students and the values presented between T0 and T1 (p = 0.022), between T1 and the evaluation moment after the CT (T2) (p = 0.003) and between T0 and T2 (p < 0.001) in the EG, and it was found that the differences have statistical significance at the level of all moments. The same situation was analyzed for the CG, with statistically significant differences being reported in the 3 moments of knowledge evaluation: T0 and T1 (p = 0.039), between T1 and T2 (p = 0.004) and between T0 and T2 (p = 0.023).

Given these results, differences in terms of classifications were analyzed, showing that the only moment in which Nursing students increased their knowledge was from T0 to T1 in the CG, with 11 participants improving their level of knowledge. The effect size was determined, obtaining results between 0.317 and 0.488 in the EG, and 0.292 and 0.402 in the CG, which indicates a medium effect size^19^.

## Discussion

This study presents a sample of Nursing students with an average age close to the mean of higher education students in Portugal, 22 years old^20^.

The participants are mostly female, just like the reality of Portuguese Nursing courses, in which 83.1% are female^21^. The same is evidenced by the data made available by the Portuguese *Ordem dos Enfermeiros*, which indicates that out of every five nurses, four are female^22^. The fact that this sample was composed of Nursing students with no clinical experience prior to the beginning of the Nursing degree course, without real experience of BLS practice, and without certified training in the scope of BLS, had as its main objective to reduce the investigation bias towards the results to be obtained regarding the variables under study.

It was also possible to verify the homogeneity of the two samples before the start of the study in terms of age, knowledge and DM, demonstrating that they have variable internal logical coherence, but with criteria of belonging to the same domain^23^.

The students’ level of knowledge about BLS is quite adequate, corroborating data from another study^24^, although in this case there were participants with experience in clinical practices and certified training in BLS.

Other studies developed identify equally positive results regarding Nursing students’ knowledge about BLS, although with lower percentages of correct answers^25^ ^)-(^ ^27^. Despite the participants’ very adequate level of knowledge about BLS, it is observed that in both research groups, overall, knowledge decreases over time, as the analysis progresses in relation to the moment before carrying out the CS scenario. This decrease in the level of knowledge about BLS is corroborated by several authors, both among students and nurses^28^ ^)-(^ ^31^.

Regarding the DM of Nursing students within the scope of BLS, it was possible to verify that the DM process of Nursing students, in terms of total medians, undergoes an improvement in EG over time, after theoretical training on BLS, skills training using HFS and carrying out CT; while in the CG the total medians suffer a decrease from the moment after carrying out theoretical training on BLS, to the moment of skills training with MFS and carrying out CT. Although among research groups higher scores were observed in the CG compared to the EG throughout the three DM evaluation moments, these data are in line with some studies found in the scientific literature, where an improvement in the DM process of Nursing students using the practice of HFS is evidenced, when compared to other types of CS^18^ ^),(^ ^32^ ^)-(^ ^33^. In the United States of America, evidence has recently emerged that the training of non-technical skills, specifically DM in Nursing students, is improved with the use of HFS, when compared to carrying out only CT practices^34^.

There were no statistically significant differences between the different moments of evaluation of each of these variables and the groups under investigation. This situation may be related to the level of knowledge that Nursing students had at the time, with the lack of previous experience in clinical practices and the lack of experience in resolving CS scenarios previously, indicating that these factors should be considered when choosing the type of CS to apply^35^ ^)-(^ ^37^.

Other studies also identify, as hypotheses of absence of statistically significant differences between the research groups and the acquisition of knowledge, the time elapsed for the practice of BLS through CS and the number of Nursing students in each CS scenario^36^.

Despite these, there are studies that document significant differences between EG and CG, when Nursing students are exposed to HFS scenario practices, compared to students who attend a theoretical training session with a case study, inserted in the TTM^32^ ^),(^ ^38^.

In terms of the DM variable, from the studies accessed, it is observed that there are some results in the opposite direction to the statements, revealing statistically significant differences between the research groups involved and the DM of Nursing students. This situation arises in a study carried out in Turkey, where the knowledge, critical thinking and DM of Nursing students faced with a situation of pre-eclampsia were analyzed, using HFS^32^, as well as in Korea, demonstrating statistically significant differences in EG’s DM capacity, after participating in the structured pre-CS preparation and briefing^18^.

In another study carried out in Oman, which involved Nursing students in the context of training skills in maternal health and obstetrics, the objective was the absence of statistically significant differences between the DM of the EG, where HFS scenarios and CT practice were applied, compared to the DM of CG students, where they only developed practice using the CT^39^. In percentage terms, the EG developed 25% of practice with HFS and 75% of practice in CT, while the CG developed 100% of its practice in CT, leading the authors to announce that the total number of hours that students have in their Nursing training in a CS context could be replaced by at least 25% with CS scenarios^39^.

In terms of longitudinal study, it is observed that the knowledge variable presented statistically significant differences in all evaluation moments, in the two research groups, allowing us to affirm that knowledge can be worked on and developed using HFS and MFS, complemented with practice in CT^18^ ^),(^ ^40^.

Although it is observed that the levels of knowledge of Nursing students about BLS decrease slightly over time, as described by several authors^28^ ^)-(^ ^30^, it is noted that they increase after theoretical training and implementation of CS in the CG, which may once again indicate suitability of the type of CS to the level of knowledge and practical experience of the participants^35^ ^)-(^ ^36^.

Regarding DM, the results indicated statistically significant differences in F1 (Definition of the Problem and Development of the Objectives) in the EG, and in F2 (Search and Data Processing) in the CG, respectively in Definition of the Problem and Development of the Objectives and Search and Data Processing. Specifically, it is observed that the HFS, together with the CT, interferes with the definition of the problem and the development of the objectives of Nursing students, with the purpose of resolving the CS scenario within the scope of BLS. Furthermore, it is also clear that the MFS enhances data search and processing in Nursing students, with a view to better DM. It should also be noted that the improvement in DM occurred in the EG with a more visible evolution between the moment before the CS scenarios were carried out and the end of the CT.

This study shows that CS is a teaching strategy suitable for DM and for improving the knowledge of Nursing students, and can replace hours in the CT context, with the existence of gains for the provision of Nursing care.

Despite the results presented, overall the DM of Nursing students from both research groups did not reveal statistically significant differences, contrary to what emerged from other studies^32^. In this sense, a possible limitation of this study is the fact that the participants at the start of the study attended the second year of the Bachelor’s degree course, at which point they had not yet had experience in CT, restricting the level of knowledge development and the DM process^35^ ^)-(^ ^36^. 

A second limitation of this study is related to the sample size (n), as occurred in another study^34^. It is worth noting that of the accessible population (N=300), 100 students volunteered (33%), but of these only 51 met the inclusion criteria defined for the study. This loss of volunteers was associated with the fact that theoretical training was carried out on weekends (a period of rest from teaching activities) and during extracurricular periods (in the separation between semesters of the academic year). This could, therefore, be a limitation that implies that the data cannot be representative of the global population, but rather of the sample under study, despite being within the sample calculation for a confidence level of 90% and a margin of error of 11%, with n equal to or greater than 48 students. 

It is therefore suggested that new experimental studies be carried out involving final-year Nursing students, with analysis of these same variables, and with a larger sample than the present study (ideally taking place during one of the working days in which there are teaching activities), in order to study the efficiency of CS compared to carrying out CT in a real environment.

## Conclusion

Carrying out this study made it possible to demonstrate that the level of knowledge of Nursing students is quite adequate in the context of BLS, but that it decreases over time, which in this study involved a period of 4 months (between the moment of the initial evaluation and the last evaluation moment), and with the lack of care practice in this same BLS scope. Furthermore, it revealed a good DM process for Nursing students through carrying out CS scenarios, demonstrating efficiency using HFS, but also with MFS. It was found that the level of knowledge and practical experience define the fidelity of CS to be applied to Nursing students.
