# Supplementary material for: Comparative study between high-fidelity simulation and medium-fidelity simulation in decision-making of nursing students: experimental study
Source: Rev Lat Am Enfermagem. 2024 Aug 12;32:e4269. doi: 10.1590/1518-8345.6847.4269 (PMC11321187; doi:10.1590/1518-8345.6847.4269)
Supplement: Translation

# Translation: Estudo comparativo entre simulação de alta-fidelidade e simulação de média-fidelidade na tomada de decisão de estudantes de enfermagem: estudo experimental*

**DOI:** 10.1590/1518-8345.6847.4270

**Authors:** Hugo Miguel Santos Duarte, Joana Antunes Castanheira, Ana Sofia Ferreira Pereira, Ângela Pragosa, Edna Tatiana Prazeres Santos, Maria dos Anjos Dixe

**Affiliations:** 1 Instituto Politécnico de Leiria, Escola Superior de Saúde de Leiria, Leiria, LEI, Portugal.; 2 Instituto Politécnico de Leiria, ciTechCare - Center for Innovative Care and Health Technology, Leiria, LEI, Portugal.; 3 Centro Hospitalar Médio Tejo, Hospital de Torres Novas, Torres Novas, TOR, Portugal.; 4 Centro Hospitalar de Leiria, Hospital de Santo André, Leiria, LEI, Portugal.

**Keywords:** Estudantes de Enfermagem, Pensamento Crítico, Raciocínio Clínico, Reanimação Cardiopulmonar, Tomada de Decisão Clínica, Treinamento com Simulação de Alta-Fidelidade

## Abstract

**Objetivo:**

comparar a tomada de decisão dos estudantes de Enfermagem, antes e após a formação teórica sobre suporte básico de vida, com recurso à prática de simulação de alta-fidelidade e simulação de média-fidelidade.

**Método:**

desenvolveu-se um estudo experimental, tipo pré e pós-teste, com análise quantitativa, descritiva e inferencial, com realização da formação teórica sobre suporte básico de vida e práticas de simulação clínica, e com avaliação dos conhecimentos e tomada de decisão dos estudantes de Enfermagem, em três momentos distintos - antes do cenário de simulação (T0), após o cenário de simulação (T1) e após a realização de ensino clínico (T2).

**Resultados:**

participaram da pesquisa 51 estudantes, com uma média de idade de 20,25±3,804, dos quais 92,2% eram do sexo feminino. Foram evidenciadas diferenças estatisticamente significativas (*F*=6,47; p=0,039) perante a definição do problema e desenvolvimento dos objetivos na tomada de decisão no grupo experimental.

**Conclusão:**

os estudantes de Enfermagem demonstram um nível de conhecimentos adequado e um bom processo de tomada de decisão, com base nos instrumentos mais atuais produzidos pela evidência científica, perante cenários de simulação clínica em suporte básico de vida, devendo esta metodologia inovadora ser aprofundada no ensino de Enfermagem.

## Destaques

**(1)** A simulação clínica promove uma boa tomada de decisão dos estudantes de Enfermagem.

**(2)** Os estudantes evidenciaram conhecimentos adequados sobre suporte básico de vida.

**(3)** Os conhecimentos e a prática são definidores da fidelidade de simulação clínica.

**(4)** O suporte básico de vida pode ser desenvolvido por simulação de alta-fidelidade.

## Introdução

O ensino de Enfermagem é fundamentalmente suportado por duas principais metodologias: Metodologia de Ensino Tradicional (MET) e Metodologia de Ensino Inovadora (MEI)^1^ ^)-(^ ^2^. A primeira baseia-se na simples transmissão de conhecimentos entre o docente e os estudantes, enquanto a MEI é caraterizada pela interação entre os conhecimentos prévios do estudante e os novos conhecimentos adquiridos, conduzindo a uma maior estabilidade cognitiva^1^ ^)-(^ ^3^.

Dentro das metodologias de ensino, existem diversas ferramentas de aprendizagem. O processo de aprendizagem em Enfermagem deve ser suportado em estratégias pedagógicas enquadradas no estatuto da centralidade do estudante, permitindo a mobilização dos seus conhecimentos prévios, não apenas assimilar os conhecimentos transmitidos, assim como potenciando o desenvolvimento da autonomia, o pensamento crítico e o julgamento clínico no processo de Tomada de Decisão (TD), salientando-se para isso a Simulação de Alta-Fidelidade (SAF)^1^ ^)-(^ ^2^ ^),(^ ^4^ ^)-(^ ^5^.

A SAF, um dos tipos de Simulação Clínica (SC) inseridos na MEI, é uma estratégia de aprendizagem que integra conhecimentos teóricos com a prática clínica, e que privilegia a reflexão e o pensamento crítico dos estudantes de Enfermagem, sendo também utilizada para estimular a análise dos problemas de múltiplas perspetivas, contribuindo para a melhoria do raciocínio clínico e do processo de TD no âmbito de competências técnicas e não técnicas^3^ ^)-(^ ^4^ ^),(^ ^6^ ^)-(^ ^10^.

A compreensão do conceito de TD é um fator importante no ensino de Enfermagem, para que possam ser desenvolvidas competências no âmbito da resolução de problemas, comunicação, priorização, raciocínio clínico, julgamento clínico e pensamento crítico^11^ ^)-(^ ^12^.

A TD clínica tem por base três requisitos fundamentais: conhecimentos na área de atuação; habilidades de pensamento; e uma perceção adequada sobre a situação ou problema^11^.

Além disso, a TD é um processo mental complexo, que deve contemplar as seguintes etapas: identificação e definição do problema; desenvolvimento de objetivos^13^; procura dos dados/fatos^12^ ^)-(^ ^13^; desenvolvimento de um modelo^13^; avaliação das alternativas e seleção da melhor solução^12^ ^)-(^ ^13^; e implementação da decisão ou planeamento do curso da ação^3^ ^),(^ ^13^. Autores^14^ afirmam ainda que o processo de TD contempla quatro fases principais: recolha de dados; processamento de dados; planeamento da ação; e implementação do plano^14^.

Dada a importância de uma adequada TD na prática clínica e a necessidade de compreender se existem diferenças no ensino de Enfermagem realizado com recurso às duas ferramentas, inseridas na MEI, com este estudo pretende-se dar resposta à questão de investigação: Existem diferenças entre a tomada de decisão dos estudantes de Enfermagem, antes e após a formação teórica sobre Suporte Básico de Vida (SBV), com recurso à prática de simulação de alta-fidelidade e simulação de média-fidelidade? Neste sentido, foi definido o seguinte objetivo principal: comparar a tomada de decisão dos estudantes de Enfermagem, antes e após a formação teórica sobre suporte básico de vida, com recurso à prática de simulação de alta-fidelidade e simulação de média-fidelidade.

## Método

### Desenho do estudo

Estudo experimental, do tipo pré e pós-teste, desenhado com grupo de controle e com cegamento, com recolha de dados realizada entre fevereiro de 2020 e fevereiro de 2021.

Para a concretização deste estudo foram consideradas variáveis que permitissem realizar uma caraterização sociodemográfica e acadêmica dos participantes (sexo, idade e ano acadêmico), e avaliassem os conhecimentos e a TD dos estudantes de Enfermagem sobre SBV. Os conhecimentos dos estudantes de Enfermagem sobre SBV foram avaliados por meio de um instrumento composto por 37 questões de resposta fechada do tipo verdadeiro ou falso, baseado em um instrumento público que permite pontuações entre 0 e 37 pontos e nas *guidelines* mais recentes sobre SBV^15^ ^)-(^ ^16^. A avaliação da TD dos participantes sobre SBV realizou-se com recurso à Escala de Tomada de Decisão Clínica em Estudantes de Enfermagem^©^ (CDMNS-PT^©^)^17^, traduzida e validada transculturalmente para os estudantes de Enfermagem portugueses, composta por 23 itens, cada um deles respondido em uma escala ordinal de frequência que varia de 1 a 5 (1-Nunca e 5-Sempre), oscilando a sua pontuação entre 23 e 115 pontos, sendo que pontuações mais elevadas traduzem índices de percepção de TD superiores. A CDMNS-PT^©^ é constituída por três fatores: Fator 1 - Definição do Problema e Desenvolvimento dos Objetivos (F1); Fator 2 - Procura e Processamento de Dados (F2); e Fator 3 - Avaliação de Alternativas, Planeamento e Implementação da Ação (F3)^17^.

### Local de estudo

O estudo foi implementado na Escola Superior de Enfermagem de Coimbra - Portugal.

### Participantes

A população acessível (N) era de 300 estudantes de Enfermagem da referida escola. Os participantes tinham como critérios de inclusão não ter experiência prévia em práticas de SAF, não ter realizado práticas clínicas, não ter prestado cuidados de saúde antes do início do curso de licenciatura, não ter formação em SBV certificada, e não ter experiência de atuação em uma situação real de SBV. Todos os estudantes que apresentaram uma resposta positiva para estas situações foram excluídos do estudo. Assim, da população acessível (N=300), voluntariaram-se 100 estudantes (33%), mas destes apenas 51 cumpriam os critérios de inclusão (Figura 1). A perda de voluntários está relacionada com o fato da formação teórica ter sido ao fim de semana, em período extracurricular.

**Figura 1:** Diagrama de fluxo CONSORT* *CONSORT = *Consolidated Standards of Reporting Trials*

Foi realizado o cálculo amostral, com base no tamanho da população (N=300), com um nível de confiança de 90% e uma margem de erro de 11%, para uma amostra igual ou superior a 48 estudantes. De referir que não existiu perda amostral entre os diferentes momentos de avaliação das variáveis.

### Intervenções

Após preenchimento do Consentimento Informado, os estudantes foram randomizados em Grupo Experimental (GE) e Grupo Controle (GC). Foi-lhes igualmente fornecido um guião de formação para estudo dos cuidados de saúde aplicados na prática de SBV. Na semana seguinte, os participantes receberam uma formação teórica sobre SBV, em período extracurricular, aproximadamente durante 45 minutos, lecionada por um Enfermeiro Especialista em Enfermagem Médico-Cirúrgica, com formação certificada em SBV pela *American Heart Association* (Figura 2).

**Figura 2:** Estudo experimental e suas intervenções

Terminada a formação teórica, os estudantes preencheram um questionário, que permitiu realizar a caraterização sociodemográfica e acadêmica, avaliar o seu nível de conhecimentos sobre SBV^15^ ^)-(^ ^16^ e avaliar a sua TD em SBV^17^, sendo este o momento de avaliação antes da realização do cenário (T0). A aplicação dos questionários foi feita por investigadores, os quais não participaram como instrutores dos cenários de SC.

Seguiu-se a divisão dos 51 estudantes de Enfermagem, respeitando a randomização realizada inicialmente, em dois grupos, com recurso a simuladores *Resusci Anne Laerdal* ^*®*^ no GE - SAF - Grupo 1, e simuladores *MegaCode Kelly Laerdal* ^*®*^ no GC - Simulação de Média-Fidelidade (SMF) - Grupo 2, e realização dos cenários sobre SBV.

O GE, antes da realização do cenário de SAF, teve um *prebriefing* com explicações sobre: o que é a SC; o que contém o laboratório de SAF; o que lhes será apresentado dentro do laboratório de SAF; e qual o objetivo de praticar o cenário de SAF. A mesma situação foi enunciada com o GC, mas neste caso sobre SMF. Em seguida, dentro de cada grupo, entraram os participantes aos pares no seu respetivo laboratório, onde dispuseram de 6 minutos para a realização do procedimento SBV, em ambiente intra-hospitalar, iniciando com um doente consciente e com dor precordial, culminando em paragem cardiorrespiratória. Ao longo dos cenários de SAF existiu um instrutor dentro do laboratório e outro dentro da sala de controle, enquanto nos cenários de SMF existiu apenas um instrutor dentro do laboratório. Logo após os cenários, desenvolveu-se uma sessão de *debriefing* reflexivo^18^ entre cada par de participantes e instrutor, e preenchimento de novo questionário, de forma a reavaliar os conhecimentos e a TD dos estudantes sobre SBV depois da conclusão dos cenários (T1). Posteriormente, desenvolveu-se o Ensino Clínico (EC), aproximadamente durante 4 meses, findo o qual os estudantes preencheram um terceiro questionário, com nova avaliação do nível de conhecimentos e TD sobre SBV, sendo este considerado o momento de avaliação após a realização do EC (T2).

### Desfechos

Os desfechos primários deste estudo previam a existência de diferenças estatisticamente significativas entre o nível de conhecimentos sobre SBV dos estudantes de Enfermagem e os grupos de investigação, assim como a existência de diferenças estatisticamente significativas entre a TD em práticas de SBV dos estudantes de Enfermagem e os grupos de investigação.

### Aleatorização

Os estudantes foram randomizados, através do sistema bola branca/bola preta, em GE e GC, formando pares dentro de cada um destes grupos atribuídos pela ordem de randomização, logo após preenchimento do Consentimento Informado, Livre e Esclarecido para a participação neste estudo.

### Cegamento

A randomização foi realizada pelo investigador principal, sendo apenas do conhecimento deste e dos instrutores envolvidos na formação prática de SBV. Nenhum dos estudantes de Enfermagem teve conhecimento da correspondência entre a bola branca/bola preta e o grupo respectivo.

### Análise dos dados

O tratamento dos dados foi realizado com recurso ao programa informático *Statistical Package for the Social Sciences*, versão 26.0. No que diz respeito às técnicas de estatística descritiva, foram calculadas frequências absolutas e relativas, medidas de tendência central e medidas de dispersão e variabilidade. Foram igualmente aplicados testes emparelhados e interdependentes não paramétricos - Mann-Whitney e Friedman. Para as provas estatísticas foram utilizados os níveis de significância: p > 0,05 - diferença não significativa; e p ≤ 0,05 - diferença significativa^19^. A interpretação dos resultados de tamanho do efeito teve em consideração os valores: r ≤ 0,2 - tamanho do efeito pequeno; 0,2 < r ≤ 0,5 - tamanho do efeito médio; 0,5 < r ≤ 1 - tamanho do efeito elevado; e r > 1 - tamanho do efeito muito elevado^19^.

### Aspectos éticos

O protocolo de investigação foi aprovado por uma Comissão de Ética em Ciências da Saúde, com o Parecer Número P.625-11/2019. Todos os dados recolhidos, após obtenção do Consentimento Informado, Livre e Esclarecido dos participantes, foram tratados com a confidencialidade e o anonimato exigidos pelo rigor e padrões da investigação.

## Resultados

Neste estudo participaram 51 estudantes de Enfermagem, com uma média de idade de 20 anos (20,3±3,8), dos quais 92,2% eram do sexo feminino. Antes da realização dos cenários de SC (T0), com recurso à estatística do teste de Levene, não foram encontradas diferenças com significado estatístico entre os GE e GC nas variáveis idade (313; p=0,786), conhecimentos (226; p=0,055) e TD (265; p=0,258), demonstrando a homogeneidade da amostra relativamente a estes dados.

Com relação à variável conhecimentos em SBV, e apesar dos estudantes apresentarem um bom nível de conhecimentos, verificamos que ao longo do tempo há um decréscimo da pontuação média em ambos os grupos do estudo (Tabela 1).

**Tabela 1:** Caracterização dos conhecimentos e tomada de decisão dos estudantes de Enfermagem e diferenças entre grupos de investigação nos três momentos de avaliação. Coimbra, Portugal, 2021

Variável			Grupo	M*	DP^†^	PM^‡^	U^§^	p^||^
**Conhecimentos**		T0^¶^	GE**	33,2	1,6	29,8	226	0,055
			GC^††^	32,3	1,5	22,0		
		T1^‡‡^	GE**	32,6	1,7	25,0	300	0,631
			GC^††^	32,7	1,6	27,0		
		T2^§§^	GE**	30,9	1,8	24,4	284,5	0,440
			GC^††^	31,3	2,1	27,6		
								
**Tomada de decisão**	T^||||^	T0^¶^	GE**	95,6	7,2	23,7	265	0,258
			GC^††^	98,3	7,9	28,4		
	F1^¶¶^		GE**	49,4	4,0	23,1	250,5	0,159
			GC^††^	51,2	4,6	29,0		
	F2***		GE**	20,7	2,5	24,5	285	0,447
			GC^††^	21,2	2,5	27,6		
	F3^†††^		GE**	25,5	2,5	24,8	294	0,555
			GC^††^	25,8	2,6	27,2		
								
	T^||||^	T1^‡‡^	GE**	95,3	8,4	23,4	258	0,206
			GC^††^	98,2	6,9	28,7		
	F1^¶¶^		GE**	49,5	4,6	23,5	260,5	0,222
			GC^††^	50,9	3,7	28,6		
	F2***		GE**	20,9	2,6	24,7	292	0,530
			GC^††^	21,4	2,5	27,3		
	F3^†††^		GE**	24,9	3,3	24,5	287	0,470
			GC^††^	25,9	2,6	27,5		
								
	T^||||^	T2^§§^	GE**	95,3	8,7	25,5	312,5	0,814
			GC^††^	95,5	7,9	26,5		
	F1^¶¶^		GE**	51,0	4,6	26,2	319	0,910
			GC^††^	50,8	4,6	25,8		
	F2***		GE**	19,8	2,8	26,9	301,5	0,655
			GC^††^	19,4	2,6	25,1		
	F3^†††^		GE**	24,5	3,9	25,0	299	0,621
			GC^††^	25,3	2,9	27,0		

*M = Média; ^†^DP = Desvio-Padrão; ^‡^PM = Posto Médio; ^§^U = Teste de Mann-Whitney; ^||^p = Significância; ^¶^T0 = Momento de avaliação antes da realização do cenário; **GE = Grupo Experimental; ^††^GC = Grupo Controle; ^‡‡^T1 = Momento de avaliação após a realização do cenário; ^§§^T2 = Momento de avaliação após a realização do ensino clínico; ^||||^T = Total do Somatório da Escala; ^¶¶^F1 = Fator 1 - Definição do Problema e Desenvolvimento dos Objetivos; ***F2 = Fator 2 - Procura e Processamento de Dados; ^†††^F3 = Fator 3 - Avaliação de Alternativas, Planeamento e Implementação da Ação

No que concerne à TD dos estudantes de Enfermagem no âmbito do SBV, variável analisada pela aplicação da CDMNS-PT^©^, observa-se uma oscilação de medianas entre 94 e 100 pontos na totalidade do instrumento, em ambos os grupos de investigação, nos três momentos de avaliação. Dentro do GE, constata-se uma manutenção do nível de TD dos estudantes de Enfermagem, neste caso com recurso à SAF, enquanto no GC, com recurso à SMF, existe um decréscimo na pontuação ao longo dos três momentos de avaliação, sendo mais evidente no terceiro momento, após a realização do EC.

Tendo em consideração os momentos de avaliação e as diferenças entre grupos, quando consideramos a variável nível de conhecimentos e TD dos estudantes de Enfermagem, observa-se que tendencialmente não existem diferenças estatisticamente significativas entre os vários momentos de avaliação de cada uma das variáveis e os grupos em investigação - GE e GC.

Em termos longitudinais, observa-se que a TD apresenta diferenças estatisticamente significativas no fator 1 *Definição do Problema e Desenvolvimento dos Objetivos*, perante o GE (p < 0,05), e no fator 2 *Procura e Processamento de Dados* no GC (p < 0,01). O valor global da TD apresenta diferenças não significativas nos dois grupos de investigação, no âmbito dos vários momentos de avaliação. Em termos do nível de conhecimentos, surgem diferenças estatisticamente significativas (p < 0,01) em ambos os grupos de investigação, entre os três momentos de avaliação, sendo mais expressivas no GE (p < 0,001) (Tabela 2).

**Tabela 2:** Resultados da aplicação do teste de Friedman sobre o nível de conhecimentos e tomada de decisão dos estudantes de Enfermagem em função do grupo de investigação nos três momentos de avaliação. Coimbra, Portugal, 2021

Grupo	Variável			PM*	M^†^	DP^‡^	*F* ^*§*^	*p* ^*||*^
**Grupo controle** (n^¶^=25)	**Conhecimento**		T0**	2,0	32,3	1,5	11,04	0,004
			T1^††^	2,4	31,3	2,0		
			T2^‡‡^	1,6	31,3	2,1		
								
	**Tomada de decisão**	T^§§^	T0**	2,2	98,3	7,9	5,71	0,058
			T1^††^	2,2	98,2	6,9		
			T2^‡‡^	1,6	95,5	7,9		
								
		F1^||||^	T0**	2,0	51,2	4,6	0,02	0,989
			T1^††^	2,0	50,9	3,7		
			T2^‡‡^	2,0	50,8	4,5		
								
		F2^¶¶^	T0**	2,2	21,2	2,5	11,04	0,004
			T1^††^	2,3	21,4	2,5		
			T2^‡‡^	1,5	19,4	2,6		
								
		F3***	T0**	2,1	25,8	2,6	0,58	0,750
			T1^††^	2,0	25,9	2,6		
			T2^‡‡^	1,9	25,3	2,9		
								
**Grupo experimental** (n^¶^=26)	**Conhecimento**		T0**	2,5	33,2	1,6	19,49	0,001
			T1^††^	2,1	31,5	1,7		
			T2^‡‡^	1,4	30,9	1,8		
								
	**Tomada de decisão**	T^§§^	T0**	2,1	95,6	7,2	0,55	0,758
			T1^††^	2,0	95,3	8,4		
			T2^‡‡^	1,9	95,3	8,7		
								
		F1^||||^	T0**	1,8	49,4	4,0	6,47	0,039
			T1^††^	1,8	49,6	4,6		
			T2^‡‡^	2,4	51,0	4,6		
		F2^¶¶^	T0**	2,2	20,7	2,5	5,61	0,061
			T1^††^	2,2	20,9	2,6		
			T2^‡‡^	1,7	19,8	2,8		
								
		F3***	T0**	2,2	25,5	2,5	1,33	0,515
			T1^††^	1,9	24,9	3,3		
			T2^‡‡^	1,9	24,5	3,9		

*PM = Posto Médio; ^†^M = Média; ^‡^DP = Desvio-Padrão; ^§^F = Teste de Friedman; ^||^p = Significância; ^¶^n = Amostra; **T0 = Momento de avaliação antes da realização do cenário; ^††^T1 = Momento de avaliação após a realização do cenário; ^‡‡^T2 = Momento de avaliação após a realização do ensino clínico; ^§§^T = Total do somatório da escala; ^||||^F1 = Fator 1 - Definição do Problema e Desenvolvimento dos Objetivos; ^¶¶^F2 = Fator 2 - Procura e Processamento de Dados; ***F3 = Fator 3 - Avaliação de Alternativas, Planeamento e Implementação da Ação

Em virtude de existirem diferenças estatisticamente significativas na Definição do Problema e Desenvolvimento dos Objetivos (F1) da variável TD, perante o GE, e na Procura e Processamento de Dados (F2), da mesma variável, perante o GC, realizou-se uma análise mais aprofundada destes fatores. Desta forma, observa-se que existem diferenças estatisticamente significativas entre o F1 da TD dos estudantes de Enfermagem do GE e o valor apresentado entre T0 e T2 (p = 0,010). No que se diz respeito ao F2 da TD, perante o GC, este também revela diferenças estatisticamente significativas nos momentos T0 e T2 (p = 0,003) e nos momentos T1 e T2 (p = 0,001) (Tabela 3).

**Tabela 3:** Resultados da aplicação do teste de classificações assinaladas por Wilcoxon sobre os momentos de avaliação dos conhecimentos e da tomada de decisão dos estudantes de Enfermagem em função do grupo de investigação. Coimbra, Portugal, 2021

Grupo	Variável			CN*	CP^†^	E^‡^	Z^§^	*p* ^*||*^	*Tamanho do Efeito*
**Grupo experimental** (n^¶^=26)	**Conhecimentos**		T0**	14	4	8	-2,29	0,022	0,317
			T1^††^						
			T1^††^	18	5	3	-3,02	0,003	0,418
			T2^‡‡^						
			T0**	21	3	2	-3,52	0,000	0,488
			T2^‡‡^						
								
**Grupo controle** (n^¶^=25)			T0**	2	11	12	-2,07	0,039	0,292
			T1^††^						
			T1^††^	16	4	5	-2,85	0,004	0,402
			T2^‡‡^						
			T0**	13	6	6	-2,27	0,023	0,322
			T2^‡‡^						
									
**Grupo experimental** (n^¶^=26)	**Tomada de decisão**	F1^§§^	T0**	10	12	4	-0,41	0,680	---
			T1^††^						
			T1^††^	7	18	1	-1,60	0,111	---
			T2^‡‡^						
			T0**	6	15	5	-2,58	0,010	0,506
			T2^‡‡^						
									
**Grupo controle** (n^¶^=25)		F2^||||^	T0**	7	8	10	-0,84	0,399	---
			T1^††^						
			T1^††^	16	3	6	-3,24	0,001	0,457
			T2^‡‡^						
			T0**	14	4	7	-3,02	0,003	0,427
			T2^‡‡^						

*CN = Classificações Negativas; ^†^CP = Classificações Positivas; ^‡^E = Empates; ^§^Z = Teste de Classificações Assinaladas por *Wilcoxon*; ^||^p = Significância; ^¶^n = Amostra; **T0 = Momento de avaliação antes da realização do cenário; ^††^T1 = Momento de avaliação após a realização do cenário; ^‡‡^T2 = Momento de avaliação após a realização do ensino clínico; ^§§^F1 = Fator 1 - Definição do Problema e Desenvolvimento dos Objetivos; ^||||^F2 = Fator 2 - Procura e Processamento de Dados

Perante estes resultados, onde foram identificados três momentos com diferenças estatisticamente significativas, dentro dos mesmos foram analisadas as melhorias que ocorreram em termos de TD dos estudantes de Enfermagem, fazendo-se denotar a evolução do T0 para o T2 do GE, com a evidência de 15 classificações com melhoria da TD. Calculou-se o tamanho do efeito, tendo-se identificado valores entre 0,427 e 0,457 na Procura e Processamento de Dados (F2), e 0,506 na Definição do Problema e Desenvolvimento dos Objetivos (F1), o que mostra um tamanho do efeito elevado neste último^19^.

Como foram identificadas diferenças no nível de conhecimentos ao longo do tempo, averiguou-se se existiam diferenças entre o total dos conhecimentos dos estudantes de Enfermagem e os valores apresentados entre T0 e T1 (p = 0,022), entre T1 e o momento de avaliação após a realização do EC (T2) (p = 0,003) e entre T0 e T2 (p < 0,001) no GE, verificando-se que as diferenças têm significado estatístico ao nível de todos os momentos. A mesma situação foi analisada para o GC, tendo sido enunciadas diferenças estatisticamente significativas nos 3 momentos de avaliação dos conhecimentos: T0 e T1 (p = 0,039), entre T1 e T2 (p = 0,004) e entre T0 e T2 (p = 0,023).

Diante destes resultados, foram analisadas as diferenças em termos de classificações, evidenciando-se que o único momento em que os estudantes de Enfermagem aumentaram os conhecimentos foi do T0 para T1 no GC, com 11 participantes a melhorarem o seu nível de conhecimentos. Determinou-se o tamanho do efeito, obtendo-se resultados entre 0,317 e 0,488 no GE, e 0,292 e 0,402 no GC, o que indica um tamanho do efeito médio^19^.

## Discussão

Este estudo apresenta uma amostra de estudantes de Enfermagem com uma idade média próxima daquela que é referida em Portugal como média dos estudantes do ensino superior, 22 anos^20^.

Os participantes são na sua maioria do sexo feminino, tal como a realidade dos cursos de Enfermagem portugueses, em que 83,1% são do sexo feminino^21^. O mesmo é enunciado pelos dados disponibilizados pela Ordem dos Enfermeiros portuguesa, que indicam que de cada cinco Enfermeiros, quatro são do sexo feminino^22^. O fato desta amostra ser composta por estudantes de Enfermagem sem experiência clínica prévia ao início do curso de licenciatura em Enfermagem, sem experiência real de prática de SBV, e sem formação certificada no âmbito de SBV, teve como objetivo principal diminuir o viés da investigação perante os resultados a obter no que concerne às variáveis em estudo.

Foi igualmente possível constatar a homogeneidade das duas amostras antes do início do estudo em termos de idade, conhecimentos e TD, demostrando possuírem coerência lógica interna variável, mas com critérios de pertença ao mesmo domínio^23^.

O nível de conhecimentos dos estudantes sobre SBV é bastante adequado, corroborando os dados de outro estudo^24^, embora nesse caso existissem participantes com experiência em práticas clínicas e formação certificada em SBV.

Outros estudos desenvolvidos identificam resultados igualmente positivos no que concerne aos conhecimentos dos estudantes de Enfermagem sobre SBV, embora com percentagens inferiores de respostas corretas^25^ ^)-(^ ^27^. Apesar do nível bastante adequado de conhecimentos dos participantes sobre SBV, observa-se que em ambos os grupos de investigação, de uma forma global, os conhecimentos diminuem com o decorrer do tempo, à medida que a análise avança em relação ao momento antes da realização do cenário de SC. Este decréscimo do nível de conhecimentos sobre SBV é corroborado por vários autores, quer em estudantes, quer em Enfermeiros^28^ ^)-(^ ^31^.

No que concerne à TD dos estudantes de Enfermagem no âmbito do SBV, foi possível constatar que o processo de TD dos estudantes de Enfermagem, em termos das medianas totais, sofre uma melhoria no GE ao longo do tempo, depois de realizada formação teórica sobre SBV, treino de competências com recurso à SAF e realização de EC; enquanto no GC as medianas totais sofrem um decréscimo do momento após a realização de formação teórica sobre SBV, para o momento de treino de competências com SMF e realização de EC. Embora entre grupos de investigação observarem-se pontuações superiores no GC comparativamente ao GE ao longo dos três momentos de avaliação da TD, estes dados estão em linha com alguns estudos encontrados na literatura científica, onde é evidenciada uma melhoria no processo de TD dos estudantes de Enfermagem com recurso à prática de SAF, quando comparado com outros tipos de SC^18^ ^),(^ ^32^ ^)-(^ ^33^. Nos Estados Unidos da América, surgiu recentemente a evidência de que o treino de competências não técnicas, especificamente da TD em estudantes de Enfermagem, é melhorado com o recurso à SAF, quando comparado com a realização apenas de práticas em EC^34^.

Não se verificaram diferenças com significado estatístico entre os distintos momentos de avaliação de cada uma dessas variáveis e os grupos em investigação. Esta situação poderá estar relacionada com o nível de conhecimentos que os estudantes de Enfermagem detinham ao momento, com a ausência de experiência prévia em práticas clínicas e a ausência de experiência na resolução de cenários de SC anteriormente, indiciando que estes fatores deverão ser considerados para a escolha do tipo de SC a aplicar^35^ ^)-(^ ^37^.

Outros estudos identificam também, como hipóteses de ausência de diferenças estatisticamente significativas entre os grupos de investigação e a aquisição de conhecimentos, o tempo decorrido para a prática de SBV através de SC e o número estudantes de Enfermagem em cada cenário de SC^36^.

Apesar destes, existem estudos que documentam diferenças significativas entre os GE e GC, quando estudantes de Enfermagem são expostos a práticas de cenários de SAF, comparativamente a estudantes que frequentam uma sessão de formação teórica com um estudo de caso, inserido na MET^32^ ^),(^ ^38^.

Em termos da variável TD, dos estudos acedidos, observa-se que existem alguns resultados no sentido contrário aos enunciados, revelando diferenças estatisticamente significativas entre os grupos de investigação envolvidos e a TD dos estudantes de Enfermagem. Esta situação surge em um estudo desenvolvido na Turquia, onde foram analisados os conhecimentos, pensamento crítico e TD dos estudantes de Enfermagem perante uma situação de pré-eclâmpsia, com recurso à SAF^32^, assim como na Coreia, com demonstração de diferenças estatisticamente significativas perante a capacidade de TD do GE, após participar na preparação pré-SC estruturada e *briefing* ^18^.

Em outro estudo realizado em Omã, que envolveu estudantes de Enfermagem no âmbito do treino de competências em saúde materna e obstetrícia, objetivou-se a ausência de diferenças estatisticamente significativas entre a TD do GE, onde foram aplicados cenários de SAF e prática de EC, comparativamente à TD dos estudantes do GC, onde apenas desenvolveram prática com recurso ao EC^39^. Em termos percentuais, o GE desenvolveu 25% de prática com SAF e 75% de prática em EC, enquanto o GC desenvolveu 100% da sua prática em EC, levando os autores a anunciar que o número total de horas que os estudantes têm na sua formação de Enfermagem em contexto de EC poderá ser substituído em pelo menos 25% por cenários de SC^39^.

Em termos de estudo longitudinal, observa-se que a variável conhecimentos apresentou diferenças estatisticamente significativas em todos os momentos de avaliação, nos dois grupos de investigação, permitindo afirmar que os conhecimentos são passíveis de ser trabalhados e desenvolvidos com recurso à SAF e à SMF, complementados com a prática realizada na forma de EC^18^ ^),(^ ^40^.

Embora se observe que os níveis de conhecimentos dos estudantes de Enfermagem sobre SBV decrescem ligeiramente ao longo do tempo, como descrito por vários autores^28^ ^)-(^ ^30^, denota-se que estes aumentam depois da realização da formação teórica e implementação de SC no GC, podendo mais uma vez indiciar adequação do tipo de SC ao nível de conhecimentos e experiência prática dos participantes^35^ ^)-(^ ^36^.

Relativamente à TD, os resultados indicaram diferenças estatisticamente significativas no F1 (Definição do Problema e Desenvolvimento dos Objetivos) no GE, e no F2 (Procura e Processamento de Dados) no GC, respetivamente na Definição do Problema e Desenvolvimento dos Objetivos e Procura e Processamento de Dados. Concretamente, observa-se que a SAF, juntamente com o EC, interfere com a definição do problema e no desenvolvimento dos objetivos dos estudantes de Enfermagem, com vistas à resolução do cenário de SC no âmbito do SBV. Para além disso, constata-se também que a SMF potencializa a procura e o processamento de dados nos estudantes de Enfermagem, com vistas a uma melhor TD. Salienta-se ainda que a melhoria da TD ocorreu no GE com uma evolução mais visível entre o momento antes da realização dos cenários de SC e o términus do EC.

Este estudo evidencia que a SC é uma estratégia de ensino adequada à TD e à melhoria dos conhecimentos dos estudantes de Enfermagem, podendo substituir horas em contexto de EC, com a existência de ganhos para a prestação de cuidados de Enfermagem.

Apesar dos resultados apresentados, na globalidade a TD dos estudantes de Enfermagem de ambos os grupos de investigação não revelou diferenças estatisticamente significativas, ao contrário do que surgiu com outros estudos^32^. Neste sentido, enuncia-se como uma possível limitação deste estudo o fato dos participantes à data de início do estudo frequentarem o segundo ano do curso de Licenciatura, momento esse em que ainda não tinham tido experiência em EC, restringindo o nível de desenvolvimento dos conhecimentos e do processo de TD^35^ ^)-(^ ^36^. 

Uma segunda limitação deste estudo está relacionada com o tamanho da amostra (n), tal como ocorreu em outro estudo^34^. É de salientar que, da população acessível (N=300), voluntariaram-se 100 estudantes (33%), mas destes apenas 51 cumpriam os critérios de inclusão definidos para o estudo. Esta perda de voluntários foi associada ao fato da formação teórica ter sido desenvolvida ao fim de semana (período de descanso das atividades letivas) e em período extracurricular (na separação entre semestres do ano letivo). Poderá, desta forma, ser uma limitação que implica que os dados possam não ser representativos da população global, mas sim da amostra em estudo, apesar de estarem dentro do cálculo amostral para um nível de confiança de 90% e uma margem de erro de 11%, com n igual ou superior a 48 estudantes. 

Sugere-se, assim, a realização de novos estudos experimentais com envolvimento de estudantes de Enfermagem finalistas, com análise destas mesmas variáveis, e com uma amostra superior à do presente estudo (idealmente a ocorrer durante um dos dias úteis em que existam atividades letivas), de forma a estudar a eficiência da SC comparativamente à realização de EC em ambiente real.

## Conclusão

A realização deste estudo permitiu evidenciar que o nível de conhecimentos dos estudantes de Enfermagem é bastante adequado no contexto de SBV, mas que diminui com o decorrer do tempo, que neste estudo envolveu um período de 4 meses (entre o momento da avaliação inicial e o último momento de avaliação), e com a inexistência de prática de cuidados neste mesmo âmbito de SBV. Para além disso, revelou um bom processo de TD dos estudantes de Enfermagem por meio da realização de cenários de SC, demonstrando eficiência com recurso à SAF, mas também com SMF. Constatou-se que o nível de conhecimentos e a experiência prática são definidores da fidelidade de SC a aplicar aos estudantes de Enfermagem.

# Translation: Estudio comparativo entre simulación de alta fidelidad y simulación de mediana fidelidad en la toma de decisiones de estudiantes de enfermería: estudio experimental*

**DOI:** 10.1590/1518-8345.6847.4268

**Authors:** Hugo Miguel Santos Duarte, Joana Antunes Castanheira, Ana Sofia Ferreira Pereira, Ângela Pragosa, Edna Tatiana Prazeres Santos, Maria dos Anjos Dixe

**Affiliations:** 1 Instituto Politécnico de Leiria, Escola Superior de Saúde de Leiria, Leiria, LEI, Portugal.; 2 Instituto Politécnico de Leiria, ciTechCare - Center for Innovative Care and Health Technology, Leiria, LEI, Portugal.; 3 Centro Hospitalar Médio Tejo, Hospital de Torres Novas, Torres Novas, TOR, Portugal.; 4 Centro Hospitalar de Leiria, Hospital de Santo André, Leiria, LEI, Portugal.

**Keywords:** Reanimación Cardiopulmonar, Enseñanza Mediante Simulación de Alta Fidelidad, Estudiantes de Enfermería, Pensamiento, Razonamiento Clínico, Toma de Decisiones Clínicas

## Abstract

**Objetivo:**

comparar la toma de decisiones de estudiantes de Enfermería, antes y después de la formación teórica sobre soporte vital básico, utilizando la práctica de simulación de alta fidelidad y simulación de mediana fidelidad.

**Método:**

se desarrolló un estudio experimental, tipo pretest y postest, con análisis cuantitativo, descriptivo e inferencial, con formación teórica sobre soporte vital básico y prácticas de simulación clínica, y con evaluación del conocimiento y la toma de decisiones de los estudiantes de Enfermería, en tres momentos distintos: antes del escenario de simulación (T0), después del escenario de simulación (T1) y después de la enseñanza clínica (T2).

**Resultados:**

participaron de la investigación 51 estudiantes, con edad promedio de 20,25±3,804 años, de los cuales 92,2% eran mujeres. Se evidenciaron diferencias estadísticamente significativas (F=6,47; p=0,039) en cuanto a la definición del problema y desarrollo de los objetivos en la toma de decisiones en el grupo experimental.

**Conclusión:**

los estudiantes de Enfermería demuestran un nivel adecuado de conocimientos y un buen proceso de toma de decisiones, basados en los instrumentos más actuales producidos por la evidencia científica, en escenarios de simulación clínica en soporte vital básico, y esta metodología innovadora debe profundizarse en la enseñanza de Enfermería.

## Destacados

**(1)** La simulación clínica promueve una buena toma de decisiones por parte de los estudiantes de Enfermería.

**(2)** Los estudiantes demostraron conocimientos adecuados sobre soporte vital básico.

**(3)** El conocimiento y la práctica definen la fidelidad de simulación clínica.

**(4)** El soporte vital básico se puede desarrollar mediante simulación de alta fidelidad.

## Introducción

La enseñanza de la Enfermería se apoya fundamentalmente en dos metodologías principales: Metodología de Enseñanza Tradicional (MET) y Metodología de Enseñanza Innovadora (MEI)^1^ ^)-(^ ^2^. La primera se basa en la simple transmisión de conocimientos entre el profesor y los estudiantes, mientras que la MEI se caracteriza por la interacción entre los conocimientos previos del alumno y los nuevos conocimientos adquiridos, dando lugar a una mayor estabilidad cognitiva^1^ ^)-(^ ^3^.

Dentro de las metodologías de enseñanza, existen varias herramientas de aprendizaje. El proceso de aprendizaje en Enfermería debe estar sustentado en estrategias pedagógicas enmarcadas en el estatuto de centralidad del estudiante, permitiendo la movilización de sus conocimientos previos, no sólo asimilar los conocimientos transmitidos, así como potenciando el desarrollo de la autonomía, el pensamiento crítico y el juicio clínico en el proceso de Toma de Decisiones (TD), destacando para este propósito la Simulación de Alta Fidelidad (SAF)^1^ ^)-(^ ^2^ ^),(^ ^4^ ^)-(^ ^5^.

La SAF, uno de los tipos de Simulación Clínica (SC) incluidos en la MEI, es una estrategia de aprendizaje que integra conocimientos teóricos con la práctica clínica, y que privilegia la reflexión y el pensamiento crítico entre los estudiantes de Enfermería, y también se utiliza para estimular el análisis de los problemas desde múltiples perspectivas, contribuyendo a la mejora del razonamiento clínico y del proceso de TD en el ámbito de las habilidades técnicas y no técnicas^3^ ^)-(^ ^4^ ^),(^ ^6^ ^)-(^ ^10^.

Comprender el concepto de TD es un factor importante en la enseñanza de Enfermería, de modo que se puedan desarrollar habilidades en los ámbitos de la resolución de problemas, comunicación, priorización, razonamiento clínico, juicio clínico y pensamiento crítico^11^ ^)-(^ ^12^.

La TD clínica se fundamenta en tres requisitos fundamentales: conocimientos en el área de actividad; habilidades de pensamiento; y una adecuada percepción de la situación o problema^11^.

Además, la TD es un proceso mental complejo, que debe incluir las siguientes etapas: identificación y definición del problema; desarrollo de objetivos^13^; búsqueda de datos/hechos^12^ ^)-(^ ^13^; desarrollo de un modelo^13^; evaluación de alternativas y selección de la mejor solución^12^ ^)-(^ ^13^; e implementación de la decisión o planificación del curso de la acción^3^ ^),(^ ^13^. Autores^14^ también afirman que el proceso de TD incluye cuatro fases principales: recolección de datos; procesamiento de datos; planificación de la acción; e implementación del plan^14^.

Dada la importancia de una adecuada TD en la práctica clínica y la necesidad de comprender si existen diferencias en la enseñanza de Enfermería realizada con dos herramientas, incluidas en la MEI, este estudio tiene como objetivo responder a la pregunta de investigación: ¿Existen diferencias entre la toma de decisiones por estudiantes de Enfermería, antes y después de la formación teórica sobre Soporte Vital Básico (SVB), utilizando la práctica de simulación de alta fidelidad y simulación de mediana fidelidad? En este sentido, se definió como objetivo principal: comparar la toma de decisiones de estudiantes de Enfermería, antes y después de la formación teórica sobre soporte vital básico, utilizando la práctica de simulación de alta fidelidad y simulación de mediana fidelidad.

## Método

### Diseño del estudio

Estudio experimental, tipo pretest y postest, diseñado con grupo control y con cegamiento, con recolección de datos realizada entre febrero de 2020 y febrero de 2021.

Para realizar este estudio se consideraron variables que permitieran una caracterización sociodemográfica y académica de los participantes (género, edad y año académico), y que evaluaran los conocimientos y la TD de los estudiantes de Enfermería sobre SVB. Los conocimientos de los estudiantes de Enfermería sobre SVB fueron evaluados mediante un instrumento compuesto por 37 preguntas de respuesta cerrada, verdadero o falso, basado en un instrumento público que permite puntuaciones entre 0 y 37 puntos y en las directrices más recientes sobre SVB^15^ ^)-(^ ^16^. La evaluación de la TD de los participantes en SVB se realizó mediante la Escala de Toma de Decisión Clínica en Estudiantes de Enfermería^©^ (CDMNS-PT^©^)^17^, traducida y validada transculturalmente para estudiantes de Enfermería portugueses, compuesta por 23 ítems, cada uno de ellos respondidos en una escala de frecuencia ordinal que varía de 1 a 5 (1-Nunca y 5-Siempre), con puntuaciones que oscilan entre 23 y 115 puntos, donde las puntuaciones más altas reflejan mayores niveles de percepción de TD. La CDMNS-PT^©^ está compuesta por tres factores: Factor 1 - Definición del Problema y Desarrollo de los Objetivos (F1); Factor 2 - Búsqueda y Procesamiento de Datos (F2); y Factor 3 - Evaluación de Alternativas, Planificación e Implementación de la Acción (F3)^17^.

### Lugar del estudio

El estudio se implementó en la *Escola Superior de Enfermagem de Coimbra* - Portugal.

### Participantes

La población accesible (N) fue de 300 estudiantes de Enfermería de dicha escuela. Los criterios de inclusión de los participantes fueron no tener experiencia previa en prácticas de SAF, no haber realizado prácticas clínicas, no haber brindado cuidados de salud antes de iniciar la carrera, no tener formación en SVB certificado, y no tener experiencia trabajando en una situación real de SVB. Todos los estudiantes que presentaron una respuesta positiva ante estas situaciones fueron excluidos del estudio. Así, de la población accesible (N=300), 100 estudiantes se ofrecieron como voluntarios (33%), pero de estos sólo 51 cumplieron los criterios de inclusión (Figura 1). La pérdida de voluntarios está relacionada con el hecho de que la formación teórica se realizó el fin de semana, durante un período extracurricular.

**Figura 1:** Diagrama de flujo CONSORT* *CONSORT = *Consolidated Standards of Reporting Trials*

El cálculo de la muestra se realizó, con base en el tamaño de la población (N=300), con un nivel de confianza del 90% y un margen de error del 11%, para una muestra igual o mayor a 48 estudiantes. Cabe señalar que no hubo pérdida de muestra entre los diferentes momentos de evaluación de las variables.

### Intervenciones

Luego de completar el Consentimiento Informado, los estudiantes fueron randomizados en Grupo Experimental (GE) y Grupo Control (GC). También se les proporcionó una guía de formación para estudio de los cuidados de salud aplicados en la práctica de SVB. La semana siguiente, los participantes recibieron una formación teórica sobre SVB, en un período extracurricular, de aproximadamente 45 minutos de duración, impartida por un Enfermero Especialista en Enfermería Médico Quirúrgica, con formación certificada en SVB por la *American Heart Association* (Figura 2).

**Figura 2:** Estudio experimental y sus intervenciones

Después de terminar la formación teórica, los estudiantes completaron un cuestionario, que permitió realizar una caracterización sociodemográfica y académica, evaluar su nivel de conocimiento sobre SVB^15^ ^)-(^ ^16^ y evaluar su TD en SVB^17^, siendo este el momento de evaluación antes de la realización del escenario (T0). Los cuestionarios fueron aplicados por investigadores, que no participaron como instructores de los escenarios de SC.

Luego, los 51 estudiantes de Enfermería fueron divididos, respetando la randomización realizada inicialmente, en dos grupos, utilizando los simuladores *Resusci Anne Laerdal* ^*®*^ en el GE - SAF - Grupo 1, y los simuladores *MegaCode Kelly Laerdal* ^*®*^ en el GC - Simulación de Mediana Fidelidad (SMF) - Grupo 2, y la realización de los escenarios sobre SVB.

El GE, antes de la realización del escenario de SAF, tuvo un *prebriefing* con explicaciones sobre: qué es la SC; qué contiene el laboratorio de SAF; qué se les presentará dentro del laboratorio de SAF; y cuál es el objetivo de practicar el escenario de SAF. La misma situación se presentó con el GC, pero en este caso sobre SMF. Luego, dentro de cada grupo, los participantes ingresaron a su respectivo laboratorio en parejas, donde tuvieron 6 minutos para realizar el procedimiento SVB, en un ambiente intrahospitalario, comenzando con un paciente consciente y con dolor en el pecho, culminando con un paro cardiorrespiratorio. En todos los escenarios de SAF hubo un instructor dentro del laboratorio y otro dentro de la sala de control, mientras que en los escenarios de SMF solo hubo un instructor dentro del laboratorio. Inmediatamente después de los escenarios, se desarrolló una sesión de *debriefing* reflexivo^18^ entre cada par de participantes y el instructor, y se completó un nuevo cuestionario, con el fin de reevaluar el conocimiento y la TD de los estudiantes sobre SVB luego de la conclusión de los escenarios (T1). Posteriormente se desarrolló la Enseñanza Clínica (EC) durante aproximadamente 4 meses, luego de la cual los estudiantes completaron un tercer cuestionario, con una nueva evaluación del nivel de conocimientos y TD sobre SVB, considerándose este el momento de evaluación posterior a la realización de la EC (T2).

### Desenlaces

Los resultados primarios de este estudio predijeron la existencia de diferencias estadísticamente significativas entre el nivel de conocimiento sobre SVB de los estudiantes de Enfermería y los grupos de investigación, así como la existencia de diferencias estadísticamente significativas entre la TD en las prácticas de SVB de los estudiantes de Enfermería y los grupos de investigación.

### Aleatorización

Los estudiantes fueron randomizados, utilizando el sistema de bola blanca/bola negra, en GE y GC, formando parejas dentro de cada uno de estos grupos asignados en el orden de randomización, inmediatamente después de completar el Consentimiento Libre, Previo e Informado para participar en este estudio.

### Cegamiento

La randomización fue realizada por el investigador principal, siendo únicamente de su conocimiento y de los instructores involucrados en la capacitación práctica de SVB. Ninguno de los estudiantes de Enfermería tenía conocimiento de la correspondencia entre la bola blanca/bola negra y el grupo respectivo.

### Análisis de los datos

El procesamiento de los datos se realizó mediante el programa informático *Statistical Package for the Social Sciences*, versión 26.0. Con respecto a las técnicas de estadística descriptiva, se calcularon frecuencias absolutas y relativas, medidas de tendencia central y medidas de dispersión y variabilidad. También se aplicaron pruebas no paramétricas pareadas e interdependientes - Mann-Whitney y Friedman. Para las pruebas estadísticas se utilizaron los niveles de significancia: p > 0,05 - diferencia no significativa; y p ≤ 0,05 - diferencia significativa^19^. La interpretación de los resultados del tamaño del efecto tuvo en cuenta los valores: r ≤ 0,2 - tamaño del efecto pequeño; 0,2 < r ≤ 0,5 - tamaño del efecto medio; 0,5 < r ≤ 1 - tamaño del efecto alto; y r > 1 - tamaño del efecto muy alto^19^.

### Aspectos éticos

El protocolo de investigación fue aprobado por un Comité de Ética en Ciencias de la Salud, con Dictamen Número P.625-11/2019. Todos los datos recolectados, luego de obtener el Consentimiento Libre, Previo e Informado de los participantes, fueron tratados con la confidencialidad y anonimato requeridos por el rigor y estándares de la investigación.

## Resultados

Participaron de este estudio 51 estudiantes de Enfermería, con edad promedio de 20 años (20,3±3,8), de los cuales 92,2% eran mujeres. Antes de realizar los escenarios de SC (T0), utilizando la estadística de la prueba de Levene, no se encontraron diferencias estadísticamente significativas entre el GE y el GC en las variables edad (313; p=0,786), conocimiento (226; p=0,055) y TD (265; p=0,258), demostrando la homogeneidad de la muestra con relación a estos datos.

En cuanto a la variable conocimientos en SVB, y a pesar de que los estudiantes presentaron un buen nivel de conocimientos, encontramos que con el tiempo hubo una disminución en el puntaje promedio en ambos grupos de estudio (Tabla 1).

**Tabla 1:** Caracterización de los conocimientos y toma de decisiones de los estudiantes de Enfermería y diferencias entre grupos de investigación en los tres momentos de evaluación. Coimbra, Portugal, 2021

Variable			Grupo	M*	DE^†^	RM^‡^	U^§^	*p* ^*||*^
**Conocimientos**		T0^¶^	GE**	33,2	1,6	29,8	226	0,055
			GC^††^	32,3	1,5	22,0		
		T1^‡‡^	GE**	32,6	1,7	25,0	300	0,631
			GC^††^	32,7	1,6	27,0		
		T2^§§^	GE**	30,9	1,8	24,4	284,5	0,440
			GC^††^	31,3	2,1	27,6		
								
**Toma de decisiones**	T^||||^	T0^¶^	GE**	95,6	7,2	23,7	265	0,258
			GC^††^	98,3	7,9	28,4		
	F1^¶¶^		GE**	49,4	4,0	23,1	250,5	0,159
			GC^††^	51,2	4,6	29,0		
	F2***		GE**	20,7	2,5	24,5	285	0,447
			GC^††^	21,2	2,5	27,6		
	F3^†††^		GE**	25,5	2,5	24,8	294	0,555
			GC^††^	25,8	2,6	27,2		
								
	T^||||^	T1^‡‡^	GE**	95,3	8,4	23,4	258	0,206
			GC^††^	98,2	6,9	28,7		
	F1^¶¶^		GE**	49,5	4,6	23,5	260,5	0,222
			GC^††^	50,9	3,7	28,6		
	F2***		GE**	20,9	2,6	24,7	292	0,530
			GC^††^	21,4	2,5	27,3		
	F3^†††^		GE**	24,9	3,3	24,5	287	0,470
			GC^††^	25,9	2,6	27,5		
	T^||||^	T2^§§^	GE**	95,3	8,7	25,5	312,5	0,814
			GC^††^	95,5	7,9	26,5		
	F1^¶¶^		GE**	51,0	4,6	26,2	319	0,910
			GC^††^	50,8	4,6	25,8		
	F2***		GE**	19,8	2,8	26,9	301,5	0,655
			GC^††^	19,4	2,6	25,1		
	F3^†††^		GE**	24,5	3,9	25,0	299	0,621
			GC^††^	25,3	2,9	27,0		

*M = Media; ^†^DE = Desviación Estándar; ^‡^RM = Rango Medio; ^§^U = Prueba de Mann-Whitney; ^||^p = Significancia; ^¶^T0 = Momento de evaluación antes de la realización del escenario; **GE = Grupo Experimental; ^††^GC = Grupo Control; ^‡‡^T1 = Momento de evaluación después de la realización del escenario; ^§§^T2 = Momento de evaluación después de la realización de la enseñanza clínica; ^||||^T = Total de la Suma de la Escala; ^¶¶^F1 = Factor 1 - Definición del Problema y Desarrollo de los Objetivos; ***F2 = Factor 2 - Búsqueda y Procesamiento de Datos; ^†††^F3 = Factor 3 - Evaluación de Alternativas, Planificación e Implementación de la Acción

Respecto a la TD de los estudiantes de Enfermería en el ámbito del SVB, variable analizada mediante la aplicación de la CDMNS-PT^©^, hay oscilación de medianas entre 94 y 100 puntos en todo el instrumento, en ambos grupos de investigación, en los tres momentos de evaluación. Dentro del GE se mantiene el nivel de la TD de los estudiantes de Enfermería, en este caso utilizando la SAF, mientras que en el GC, utilizando la SMF, hay una disminución en la puntuación a lo largo de los tres momentos de evaluación, siendo más evidente en el tercer momento, tras realizar la EC.

Teniendo en cuenta los momentos de evaluación y las diferencias entre grupos, cuando consideramos la variable nivel de conocimientos y TD de los estudiantes de Enfermería, se observa que no suelen existir diferencias estadísticamente significativas entre los distintos momentos de evaluación de cada una de las variables y los grupos de investigación - GE y GC.

En términos longitudinales, se observa que la TD presenta diferencias estadísticamente significativas en el factor 1 *Definición del Problema y Desarrollo de los Objetivos*, respecto al GE (p < 0,05), y en el factor 2 *Búsqueda y Procesamiento de Datos* en el GC (p < 0,01). El valor global de la TD presenta diferencias no significativas en los dos grupos de investigación, en el ámbito de los distintos momentos de evaluación. En cuanto al nivel de conocimientos, surgen diferencias estadísticamente significativas (p < 0,01) en ambos grupos de investigación, entre los tres momentos de evaluación, siendo más significativas en el GE (p < 0,001) (Tabla 2).

**Tabla 2:** Resultados de la aplicación de la prueba de Friedman sobre el nivel de conocimientos y toma de decisiones de los estudiantes de Enfermería según el grupo de investigación en los tres momentos de evaluación. Coimbra, Portugal, 2021

Grupo	Variable			RM*	M^†^	DE^‡^	*F* ^*§*^	*p* ^*||*^
**Grupo control** (n^¶^=25)	**Conocimientos**		T0**	2,0	32,3	1,5	11,04	0,004
			T1^††^	2,4	31,3	2,0		
			T2^‡‡^	1,6	31,3	2,1		
							
	**Toma de decisiones**	T^§§^	T0**	2,2	98,3	7,9	5,71	0,058
			T1^††^	2,2	98,2	6,9		
			T2^‡‡^	1,6	95,5	7,9		
						
		F1^||||^	T0**	2,0	51,2	4,6	0,02	0,989
			T1^††^	2,0	50,9	3,7		
			T2^‡‡^	2,0	50,8	4,5		
						
		F2^¶¶^	T0**	2,2	21,2	2,5	11,04	0,004
			T1^††^	2,3	21,4	2,5		
			T2^‡‡^	1,5	19,4	2,6		
						
		F3***	T0**	2,1	25,8	2,6	0,58	0,750
			T1^††^	2,0	25,9	2,6		
			T2^‡‡^	1,9	25,3	2,9		
**Grupo experimental** (n^¶^=26)	**Conocimientos**		T0**	2,5	33,2	1,6	19,49	0,001
			T1^††^	2,1	31,5	1,7		
			T2^‡‡^	1,4	30,9	1,8		
							
	**Toma de decisiones**	T^§§^	T0**	2,1	95,6	7,2	0,55	0,758
			T1^††^	2,0	95,3	8,4		
			T2^‡‡^	1,9	95,3	8,7		
						
		F1^||||^	T0**	1,8	49,4	4,0	6,47	0,039
			T1^††^	1,8	49,6	4,6		
			T2^‡‡^	2,4	51,0	4,6		
						
		F2^¶¶^	T0**	2,2	20,7	2,5	5,61	0,061
			T1^††^	2,2	20,9	2,6		
			T2^‡‡^	1,7	19,8	2,8		
						
		F3***	T0**	2,2	25,5	2,5	1,33	0,515
			T1^††^	1,9	24,9	3,3		
			T2^‡‡^	1,9	24,5	3,9		

*RM = Rango Medio; ^†^M = Media; ^‡^DE = Desviación Estándar; ^§^F = Prueba de Friedman; ^||^p = Significancia; ^¶^n = Muestra; **T0 = Momento de evaluación antes de la realización del escenario; ^††^T1 = Momento de evaluación después de la realización del escenario; ^‡‡^T2 = Momento de evaluación después de la realización de la enseñanza clínica; ^§§^T = Total de la suma de la escala; ^||||^F1 = Factor 1 - Definición del Problema y Desarrollo de los Objetivos; ^¶¶^F2 = Factor 2 - Búsqueda y Procesamiento de Datos; ***F3 = Factor 3 - Evaluación de Alternativas, Planificación e Implementación de la Acción

Debido a la existencia de diferencias estadísticamente significativas en la Definición del Problema y Desarrollo de los Objetivos (F1) de la variable TD, frente al GE, y en la Búsqueda y Procesamiento de Datos (F2), de la misma variable, frente al GC, se realizó un análisis más profundo de estos factores. De esta manera, se observa que existen diferencias estadísticamente significativas entre el F1 de la TD de los estudiantes de Enfermería del GE y el valor presentado entre T0 y T2 (p = 0,010). En cuanto al F2 de la TD, respecto al GC, también revela diferencias estadísticamente significativas en los momentos T0 y T2 (p = 0,003) y en los momentos T1 y T2 (p = 0,001) (Tabla 3).

**Tabla 3:** Resultados de la aplicación de la prueba de rangos con signo de Wilcoxon en los momentos de evaluación de los conocimientos y de la toma de decisiones de los estudiantes de Enfermería según el grupo de investigación. Coimbra, Portugal, 2021

Grupo	Variable			CN*	CP^†^	E^‡^	Z^§^	*p* ^*||*^	*Tamaño del efecto*
**Grupo experimental** (n^¶^=26)	**Conocimientos**		T0**	14	4	8	-2,29	0,022	0,317
			T1^††^						
			T1^††^	18	5	3	-3,02	0,003	0,418
			T2^‡‡^						
			T0**	21	3	2	-3,52	0,000	0,488
			T2^‡‡^						
								
**Grupo control** (n^¶^=25)			T0**	2	11	12	-2,07	0,039	0,292
			T1^††^						
			T1^††^	16	4	5	-2,85	0,004	0,402
			T2^‡‡^						
			T0**	13	6	6	-2,27	0,023	0,322
			T2^‡‡^						
									
**Grupo experimental** (n^¶^=26)	**Toma de decisiones**	F1^§§^	T0**	10	12	4	-0,41	0,680	---
			T1^††^						
			T1^††^	7	18	1	-1,60	0,111	---
			T2^‡‡^						
			T0**	6	15	5	-2,58	0,010	0,506
			T2^‡‡^						
								
**Grupo control**(n^¶^=25)		F2^||||^	T0**	7	8	10	-0,84	0,399	---
			T1^††^						
			T1^††^	16	3	6	-3,24	0,001	0,457
			T2^‡‡^						
			T0**	14	4	7	-3,02	0,003	0,427
			T2^‡‡^						

*CN = Clasificaciones Negativas; ^†^CP = Clasificaciones Positivas; ^‡^E = Empates; ^§^Z = Prueba de Rangos con Signo de Wilcoxon; ^||^p = Significancia; ^¶^n = Muestra; **T0 = Momento de evaluación antes de la realización del escenario; ^††^T1 = Momento de evaluación después de la realización del escenario; ^‡‡^T2 = Momento de evaluación después de la realización de la enseñanza clínica; ^§§^F1 = Factor 1 - Definición del Problema y Desarrollo de los Objetivos; ^||||^F2 = Factor 2 - Búsqueda y Procesamiento de Datos

A la vista de estos resultados, donde se identificaron tres momentos con diferencias estadísticamente significativas, dentro de ellos se analizaron las mejoras ocurridas en cuanto a la TD de los estudiantes de Enfermería, denotando la evolución del T0 al T2 del GE, con la evidencia de 15 clasificaciones con mejora de la TD. Se calculó el tamaño del efecto, identificándose valores entre 0,427 y 0,457 en la Búsqueda y Procesamiento de Datos (F2), y 0,506 en la Definición de Problemas y Desarrollo de los Objetivos (F1), lo que resalta un alto tamaño del efecto en este último^19^.

A medida que se identificaron diferencias en el nivel de conocimientos a lo largo del tiempo, se verificó si existían diferencias entre el conocimiento total de los estudiantes de Enfermería y los valores presentados entre T0 y T1 (p = 0,022), entre T1 y el momento de la evaluación después de la EC (T2) (p = 0,003) y entre T0 y T2 (p < 0,001) en el GE, habiéndose comprobado que las diferencias tienen significación estadística a nivel de todos los momentos. La misma situación se analizó para el GC, reportándose diferencias estadísticamente significativas en los 3 momentos de evaluación de los conocimientos: T0 y T1 (p = 0,039), entre T1 y T2 (p = 0,004) y entre T0 y T2 (p = 0,023).

Ante estos resultados, se analizaron las diferencias en cuanto a las clasificaciones, mostrando que el único momento en que los estudiantes de Enfermería aumentaron sus conocimientos fue del T0 al T1 en el GC, siendo 11 participantes mejorando su nivel de conocimientos. Se determinó el tamaño del efecto, obteniendo resultados entre 0,317 y 0,488 en el GE, y 0,292 y 0,402 en el GC, lo que indica un tamaño del efecto medio^19^.

## Discusión

Este estudio presenta una muestra de estudiantes de Enfermería con una edad media cercana a la media de los estudiantes de educación superior en Portugal, 22 años^20^.

Los participantes son mayoritariamente mujeres, como en la realidad de los cursos de Enfermería portugueses, donde el 83,1% son mujeres^21^. Lo mismo se desprende de los datos facilitados por la *Ordem dos Enfermeiros* portuguesa, que indican que de cada cinco enfermeras, cuatro son mujeres^22^. El hecho de que esta muestra estuviera compuesta por estudiantes de Enfermería sin experiencia clínica previa al inicio de la carrera de Enfermería, sin experiencia real en la práctica de SVB y sin formación certificada en el ámbito de SVB, tuvo como principal objetivo reducir el sesgo de investigación hacia los resultados a obtener respecto de las variables en estudio.

También se pudo verificar la homogeneidad de las dos muestras antes del inicio del estudio en términos de edad, conocimientos y TD, demostrando que tienen coherencia lógica interna variable, pero con criterios de pertenencia al mismo dominio^23^.

El nivel de conocimiento de los estudiantes sobre SVB es bastante adecuado, corroborando datos de otro estudio^24^, aunque en este caso hubo participantes con experiencia en prácticas clínicas y formación certificada en SVB.

Otros estudios desarrollados identifican resultados igualmente positivos en cuanto al conocimiento de los estudiantes de Enfermería sobre SVB, aunque con porcentajes menores de respuestas correctas^25^ ^)-(^ ^27^. A pesar del muy adecuado nivel de conocimiento de los participantes sobre SVB, se observa que, en ambos grupos de investigación, en general, el conocimiento disminuye con el tiempo, a medida que avanza el análisis en relación al momento anterior a la realización del escenario de SC. Esta disminución en el nivel de conocimientos sobre SVB es corroborada por varios autores, tanto entre estudiantes como entre enfermeros^28^ ^)-(^ ^31^.

Respecto a la TD de los estudiantes de Enfermería en el ámbito del SVB, se pudo verificar que el proceso de TD de los estudiantes de Enfermería, en términos de medianas totales, sufre una mejora en el GE en el tiempo, luego de la formación teórica en SVB, la formación de habilidades utilizando la SAF y realización de EC; mientras que en el GC las medianas totales sufren un descenso desde el momento posterior a la realización de la formación teórica sobre SVB, hasta el momento de la formación de habilidades con SMF y realización de EC. Si bien entre los grupos de investigación se observaron puntuaciones más altas en el GC en comparación con el GE a lo largo de los tres momentos de evaluación de la TD, estos datos están en línea con algunos estudios encontrados en la literatura científica, donde se evidencia una mejora en el proceso de TD de los estudiantes de Enfermería utilizando la práctica de SAF, en comparación con otros tipos de SC^18^ ^),(^ ^32^ ^)-(^ ^33^. En Estados Unidos de América recientemente ha surgido evidencia de que la formación de habilidades no técnicas, específicamente TD en estudiantes de Enfermería, se mejora con el uso de la SAF, en comparación con la realización únicamente de prácticas de EC^34^.

No hubo diferencias estadísticamente significativas entre los distintos momentos de evaluación de cada una de estas variables y los grupos investigados. Esta situación puede estar relacionada con el nivel de conocimientos que tenían los estudiantes de Enfermería en ese momento, con la falta de experiencia previa en prácticas clínicas y la falta de experiencia en la resolución de escenarios de SC anteriormente, lo que indica que estos factores deben ser considerados al momento de elegir el tipo de SC a ser aplicada^35^ ^)-(^ ^37^.

Otros estudios también identifican, como hipótesis de ausencia de diferencias estadísticamente significativas entre los grupos de investigación y la adquisición de conocimientos, el tiempo transcurrido para la práctica de SVB a través de SC y el número de estudiantes de Enfermería en cada escenario de SC^36^.

A pesar de esto, existen estudios que documentan diferencias significativas entre GE y GC, cuando los estudiantes de Enfermería son expuestos a prácticas de escenarios de SAF, en comparación con los estudiantes que asisten a una sesión de formación teórica con un estudio de caso, insertado en la MET^32^ ^),(^ ^38^.

En cuanto a la variable TD, de los estudios accedidos, se observa que existen algunos resultados en sentido contrario a lo afirmado, revelando diferencias estadísticamente significativas entre los grupos de investigación involucrados y la TD de los estudiantes de Enfermería. Esta situación surge en un estudio realizado en Turquía, donde se analizaron, mediante SAF, los conocimientos, el pensamiento crítico y la TD de los estudiantes de Enfermería ante una situación de preeclampsia^32^, así como en Corea, con demostración de diferencias estadísticamente significativas en la capacidad de TD del GE, después de participar en la preparación previa a la SC estructurada y *briefing* ^18^.

En otro estudio realizado en Omán, que involucró a estudiantes de Enfermería en el contexto de la formación de habilidades en salud materna y obstetricia, el objetivo fue la ausencia de diferencias estadísticamente significativas entre la TD del GE, donde se aplicaron escenarios de SAF y la práctica de EC, comparada a la TD de estudiantes del GC, donde solo desarrollaron práctica utilizando la EC^39^. En términos porcentuales, el GE desarrolló el 25% de su práctica con SAF y el 75% de su práctica en EC, mientras que el GC desarrolló el 100% de su práctica en EC, lo que llevó a los autores a anunciar que el número total de horas que tienen los estudiantes en su formación de Enfermería en contexto de EC podría sustituirse en al menos un 25% por escenarios de SC^39^.

En términos de estudio longitudinal, se observa que la variable conocimientos presentó diferencias estadísticamente significativas en todos los momentos de evaluación, en los dos grupos de investigación, lo que permite afirmar que los conocimientos pueden ser trabajados y desarrollados utilizando SAF y SMF, complementados con la práctica realizada en forma de EC^18^ ^),(^ ^40^.

Si bien se observa que los niveles de conocimientos de los estudiantes de Enfermería sobre SVB disminuyen levemente con el tiempo, como lo describen varios autores^28^ ^)-(^ ^30^, se observa que estos aumentan después de la formación teórica y la implementación de SC en el GC, lo que puede indicar una vez más la adecuación del tipo de SC al nivel de conocimientos y experiencia práctica de los participantes^35^ ^)-(^ ^36^.

Con relación a la TD, los resultados indicaron diferencias estadísticamente significativas en el F1 (Definición del Problema y Desarrollo de los Objetivos) en el GE, y en el F2 (Búsqueda y Procesamiento de Datos) en el GC, respectivamente en la Definición del Problema y Desarrollo de los Objetivos y Búsqueda y Procesamiento de Datos. Específicamente, se observa que la SAF, junto con la EC, interfiere en la definición del problema y en el desarrollo de los objetivos de los estudiantes de Enfermería, con el fin de resolver el escenario de SC en el ámbito del SVB. Además, también es claro que la SMF potencia la búsqueda y procesamiento de datos en estudiantes de Enfermería, con miras a una mejor TD. Cabe señalar también que la mejora de la TD se produjo en el GE con una evolución más visible entre el momento previo a la realización de los escenarios de SC y el final de la EC.

Este estudio muestra que la SC es una estrategia de enseñanza adecuada para la TD y para la mejora de los conocimientos de los estudiantes de Enfermería, pudiendo sustituir horas en el contexto de la EC, con ganancias para la prestación de cuidados de Enfermería.

A pesar de los resultados presentados, en general la TD de los estudiantes de Enfermería de ambos grupos de investigación no reveló diferencias estadísticamente significativas, contrariamente a lo que surge de otros estudios^32^. En este sentido, una posible limitación de este estudio es el hecho de que los participantes al inicio del estudio cursaban el segundo año de la carrera, momento en el que aún no tenían experiencia en EC, restringiendo el nivel de desarrollo de los conocimientos y del proceso de TD^35^ ^)-(^ ^36^. 

Una segunda limitación de este estudio está relacionada con el tamaño de la muestra (n), como ocurrió en otro estudio^34^. Cabe señalar que, de la población accesible (N=300), 100 estudiantes se ofrecieron como voluntarios (33%), pero de estos sólo 51 cumplieron con los criterios de inclusión definidos para el estudio. Esta pérdida de voluntarios estuvo asociada a que la formación teórica se realizaba los fines de semana (período de descanso de las actividades lectivas) y en periodo extracurricular (en la separación entre semestres del año académico). Esto podría, por tanto, ser una limitación que implique que los datos puedan no ser representativos de la población global, sino de la muestra objeto de estudio, a pesar de estar dentro del cálculo muestral para un nivel de confianza del 90% y un margen de error del 11%, con n igual o mayor a 48 estudiantes. 

Por lo tanto, se sugiere realizar nuevos estudios experimentales involucrando estudiantes de último año de Enfermería, analizando estas mismas variables, y con una muestra mayor que la del presente estudio (idealmente durante uno de los días hábiles en que hay actividades lectivas), con el fin de estudiar la eficiencia de la SC en comparación con la realización de EC en un ambiente real.

## Conclusión

La realización de este estudio permitió demostrar que el nivel de conocimientos de los estudiantes de Enfermería es bastante adecuado en el contexto de SVB, pero que disminuye con el tiempo, que en este estudio implicó un período de 4 meses (entre el momento de la evaluación inicial y el último momento de evaluación), y con la falta de práctica de cuidados en este mismo ámbito de SVB. Además, reveló un buen proceso de TD de los estudiantes de Enfermería mediante la realización de escenarios de SC, demostrando eficiencia utilizando la SAF, pero también con SMF. Se encontró que el nivel de conocimientos y la experiencia práctica definen la fidelidad de SC a ser aplicada a los estudiantes de Enfermería.
